# Mapping clinical reasoning literature across the health professions: a scoping review

**DOI:** 10.1186/s12909-020-02012-9

**Published:** 2020-04-07

**Authors:** Meredith E. Young, Aliki Thomas, Stuart Lubarsky, David Gordon, Larry D. Gruppen, Joseph Rencic, Tiffany Ballard, Eric Holmboe, Ana Da Silva, Temple Ratcliffe, Lambert Schuwirth, Valérie Dory, Steven J. Durning

**Affiliations:** 1 Institute of Health Sciences Education in the Faculty of Medicine, McGill University, Room 200 Lady Meredith House, 1110 Pine Avenue West, Montreal, QC H3A 1A3 Canada; 2 School of Physical and Occupational Therapy, Institute of Health Sciences Education in the Faculty of Medicine at McGill University, Centre for Interdisciplinary Research in Rehabilitation of greater Montreal, Montréal, Canada; 3 Department of Neurology and Institute of Health Sciences Education, McGill University, Montreal, Canada; 4 Division of Emergency Medicine and the Department of Surgery, Duke University School of Medicine, Durham, North Carolina USA; 5 Department of Learning Health Sciences, University of Michigan Medical School, Ann Arbor, USA; 6 Division of General Internal Medicine at Tufts Medical Center, Tufts University School of Medicine, Boston, MA USA; 7 University of Michigan, Ann Arbor, MI USA; 8 Chief Research, Milestone Development, and Evaluation Officer, ACGME, Chicago, IL USA; 9 Feinberg School of Medicine of Northwestern University, Chicago, IL USA; 10 Swansea University Medical School, Swansea, UK; 11 Department of Medicine, University of Texas Health Science Center, San Antonio, TX USA; 12 Flinders University, Adelaide, Australia; 13 Maastricht University, Maastricht, Netherlands; 14 Chang Gung University, Taoyuan City, Taiwan; 15 Uniformed Services University of the Health Sciences, Bethesda, USA; 16 Department of Medicine, an Assessment Specialist for undergraduate medical education in the Faculty of Medicine, McGill University, Montreal, Canada; 17 Department of Medicine, Uniformed Services University of the Health Sciences, Bethesda, MD USA

**Keywords:** Clinical reasoning, Health professions, Synthesis, Scoping review, Teaching, Assessment, Education

## Abstract

**Background:**

Clinical reasoning is at the core of health professionals’ practice. A mapping of what constitutes clinical reasoning could support the teaching, development, and assessment of clinical reasoning across the health professions.

**Methods:**

We conducted a scoping study to map the literature on clinical reasoning across health professions literature in the context of a larger Best Evidence Medical Education (BEME) review on clinical reasoning assessment. Seven databases were searched using subheadings and terms relating to clinical reasoning, assessment, and Health Professions. Data analysis focused on a comprehensive analysis of bibliometric characteristics and the use of varied terminology to refer to clinical reasoning.

**Results:**

Literature identified: 625 papers spanning 47 years (1968–2014), in 155 journals, from 544 first authors, across eighteen Health Professions. Thirty-seven percent of papers used the term clinical reasoning; and 110 other terms referring to the concept of clinical reasoning were identified. Consensus on the categorization of terms was reached for 65 terms across six different categories: reasoning skills, reasoning performance, reasoning process, outcome of reasoning, context of reasoning, and purpose/goal of reasoning. Categories of terminology used differed across Health Professions and publication types.

**Discussion:**

Many diverse terms were present and were used differently across literature contexts. These terms likely reflect different operationalisations, or conceptualizations, of clinical reasoning as well as the complex, multi-dimensional nature of this concept. We advise authors to make the intended meaning of ‘clinical reasoning’ and associated terms in their work explicit in order to facilitate teaching, assessment, and research communication.

## Background

Clinical reasoning has been called the backbone of clinical practice [1, 2]. Competency frameworks across the Health Professions (e.g. Accreditation Council for Graduate Medical Education Core Competencies, the Royal College of Physicians and Surgeons of Canada’s CanMEDS framework, the General Medical Council’s Good Medical Practice, the Canadian Association of Occupational Therapists’ Profile of Practice, the Canadian Physiotherapy Association Competency Profile) [3–7] highlight the importance of clinical reasoning. Implementing these policy documents and frameworks in the training of health professionals requires a clear conceptualization of clinical reasoning to support its assessment and teaching.

While considered core to the practice of health professionals [1, 8], clinical reasoning has been discussed as either a multifaceted construct [9, 10] or a ‘black box’ phenomenon [11]. In broad terms, clinical reasoning reflects the thinking or reasoning that a health practitioner engages in to solve and manage a clinical problem. The field of clinical reasoning research represents a large literature that is rooted in early work by Elstein [12], Barrows [13, 14], Feltovitch [14], Neufeld [15], Schmidt [16], and Norman [17, 18], with a heavy focus on characterizing the cognitive processes that underpin clinical reasoning. Since then, clinical reasoning has been variably described as a process or an outcome [19]; has been discussed through the lens of various frameworks [20]; and interpreted for multiple audiences—from scholars to clinical teachers [19]. This broad and substantive literature notwithstanding, little consensus exists regarding the definition of clinical reasoning [20].

One recent review considered clinical reasoning through a series of different conceptual lenses [20], and other recent work offered insights into how various theories of clinical reasoning may be reflected in current teaching and assessment practices [21]. These works, however, are limited to the field of medicine, and are not the result of a systematic investigation of the literature across Health Professions. Given current emphasis on interprofessional training [22], and the thread of clinical reasoning throughout health professions competency profiles [3–7], a careful mapping of the concept of clinical reasoning across professions is necessary to support both profession-specific and interprofessional learning, assessment, and research. Here, we report on a scoping review conducted with the support of the Best Evidence Medical Education (BEME) collaboration [23] with the purpose of answering the question “How is clinical reasoning described in the Health Professions Education (HPE) literature?”

## Methods

### Scoping methodology

Due to the exploratory nature of this project, and the breadth of the potentially relevant literature, we chose a scoping review methodology for this project. Scoping reviews are increasingly used in Health Professions Education (HPE) to synthesize and map diverse bodies of literature in both well-defined and emerging domains. Further details regarding scoping reviews in HPE can be found in Thomas et al. [24, 25]. Scoping methodology allows for the inclusion and synthesis of various types of literature (e.g. review articles, primary work, commentaries and editorials), methodological approaches (e.g. experimental designs, descriptive studies, ethnographic studies), and data analysis approaches (qualitative, quantitative, or mixed approaches). Scoping reviews do not necessitate a formal quality appraisal of the literature [25, 26] and given the inclusion of various literature types in the current review (e.g. primary literature and commentaries), with the focus on descriptions of clinical reasoning, we judged that a quality appraisal was not appropriate nor would it add meaningfully to the results of our review.

### Study design

Mapping is defined as a process whereby the identified literature is represented both numerically (quantitatively) and thematically (qualitatively). Our specific methods aligned with the 5-step methodological framework recommended by Arksey and O’Malley, and are presented below [26].

- Step 1: Identification of a research question.

The question guiding this review was “How is clinical reasoning described in the Health Professions Education (HPE) literature?”

- Step 2: Identifying relevant research studies.

The review described in this paper is one component of a larger Best Evidence Medical Education (BEME) commissioned synthesis on assessment of clinical reasoning (for information on BEME, please see: www.bemecollaboration.org). Our scoping review draws on literature identified through the larger review [27] (reflected in the search strategy in Additional file 1: Appendix 1); however, study inclusion, data extraction, and analysis were conducted independently. Between 2013 and 2014, the team worked with a librarian to design a search comprised of three constructs: HPE, clinical reasoning, and assessment. Each article captured by the search included search terms or subheadings related to all three constructs (i.e. any given paper identified by the search would include a health profession, in an educational or assessment context, with some mention of the construct of clinical reasoning). The search strategy was vetted by two other academic health sciences librarians, adapted to the following databases: MEDLINE, ERIC, CINHAL, PsychINFO, Scopus, Google Scholar, and New York Academy of Medicine (NYAM) Grey Literature Report; and restricted to English-language papers.

- Step 3: Study selection.

**Fig. 1:**
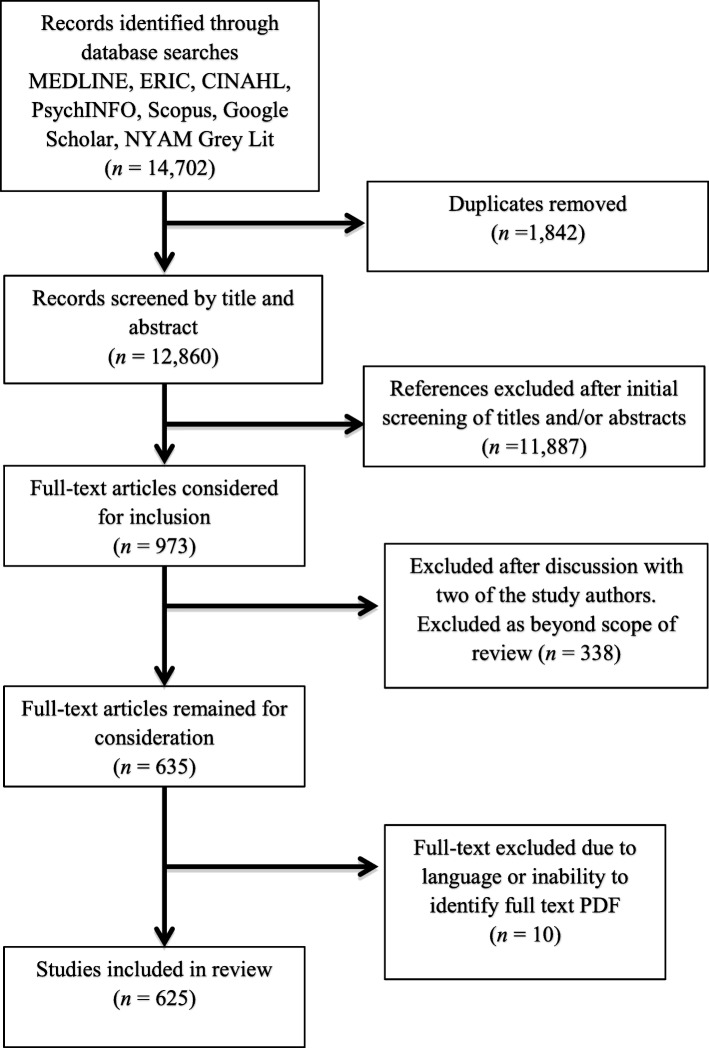
PRISMA Flow-chart of article selection^27^

Articles identified by the search strategy were screened by the larger Assessment Review Team [27], relying primarily on title and abstract review. In addition to selecting articles relevant to the review of assessments of clinical reasoning, [27] reviewers were asked to identify articles relevant for a review of the definitions of clinical reasoning; more specifically, identifying papers that either contained a definition of clinical reasoning, an associated term, or could contribute to understanding how clinical reasoning is defined in the literature. Reviewers identified 635 articles (625 of which were in English with full-text available) as relevant to the definitional review (Fig. 1). Given the large number of remaining papers identified by the Assessment Review Team, we engaged in an additional round of inclusion to ensure papers identified would contribute meaningfully to our scoping review. During this secondary round, six pairs of reviewers reviewed a total of 7 papers each (7% of database) to reassess whether each paper should be included based on the goals of the review. Initial agreement regarding inclusion within pairs of reviewers was unexpectedly low (ranging from 14 to 71%). We hypothesized that the lack of agreement was in part due to divergent conceptualizations of clinical reasoning within our own team (Young et al) [28].

In response to these findings, we paused the review process and engaged in a reflective exercise in which each team member answered questions regarding their definition of clinical reasoning and component processes. This exercise, the findings of which are reported elsewhere (Young et al) [28], revealed variation within the team regarding what was considered as ‘relevant’ contributors to clinical reasoning. As the purpose of the current review was to map the breadth of the literature, we proceeded with the review following team discussion and erred on the side of inclusion, extracting data from all 625 previously identified articles.

- Step 4: Charting the data.

The data collection tool (Additional file 1: Appendix 2) used in this review was developed using a multistep iterative process with two rounds of revision followed by usability testing. We piloted the original extraction form with the review team (*n* = 12 individuals working in teams of two), established reasonable agreement on co-extracted data on quantitative extraction items, and refined it based on usability ratings and team member suggestions.

A second phase of co-coding and data extractions occurred with the revised tool (Additional file 1: Appendix 2). Six pairs of reviewers extracted seven papers each, for a total of 42 papers (another 7% of the database). Given that several of the extraction items depended on the coder to apply their knowledge and interpret findings within the papers, and given the multiple perspectives within our review team [27], data was extracted using open-ended items to allow for interpretation and flexibility (Additional file 1: Appendix 2). Given the importance of diversity for our attempt to map the breadth of the literature, reaching agreement was not our aim. Therefore, we proceeded with single coders (*n* = 13) for the remainder of the database. We used DistillerSR software (Evidence Partners, Ottawa, Canada) for data extraction and database management, Excel (Microsoft Excel 2013, Redmond, Washington, U.S) and Prism (Prism GraphPad Software, Inc., La Jolla, CA, USA) for analysis and graphic representations.

- Step 5: Collating, summarizing and presenting findings.

### Description of analytical process

We used several approaches to summarize our study findings. In this paper, we focus on a multi-dimensional description of the database that formed the foundation of this project. To characterize the articles included in this review we focused on: profession represented (e.g. nursing, medicine, physical therapy), learner level (e.g. undergraduate, postgraduate), paper type (e.g. commentary, original research, review), country of origin, the presence of the term ‘clinical reasoning’, and other terms used to refer to clinical reasoning (when appropriate).

Terminology used to refer to clinical reasoning: For each paper, team members were asked to identify whether the term clinical reasoning was used (yes/no), and whether any other term was used to refer to clinical reasoning within the text. Team members could identify up to three terms per text, relying on their content expertise to determine relevance of a given term. Few constraints were given to the team, and team members were encouraged to apply their own conceptualizations of clinical reasoning during extraction [28]. Terms identified (*n* = 110) that were used interchangeably with clinical reasoning (e.g. diagnostic reasoning) were then iteratively coded. First, MY engaged in an inductive categorization of terms, informed by her knowledge of the clinical reasoning literature. This initial category structure was critically revised by AT and SL and adapted iteratively. Following refinement of the categories of terms, LG and DG reviewed the category labels, the identified terms, and assigned each term to a single category independently. Following this, MY, LG and DG discussed the process, reviewed their categorization of terms, and decided whether they would continue to assign a given term to a certain category or revisit their categorization. This process resulted in the team agreeing on the categorization of 65 (59%) terms across 6 categories. Terms for which the team could not agree were not included in the analyses reported in this manuscript. This categorization process is described in more detail elsewhere (Young et al. 2019) [29], including the terms for which consensus was not possible.

Exploration of terminology across publication characteristics: Whether or not a publication used the term ‘clinical reasoning’, and the categories of terminology other than ‘clinical reasoning’ were used to explore how these different categories of terms were used across articles included in this study. Analysis explored the distribution of these different categories of terms across different Health Professions, different publication types, and papers that included (or not) an assessment of clinical reasoning.

## Results

### Nature and distribution of the studies

**Fig. 2:**
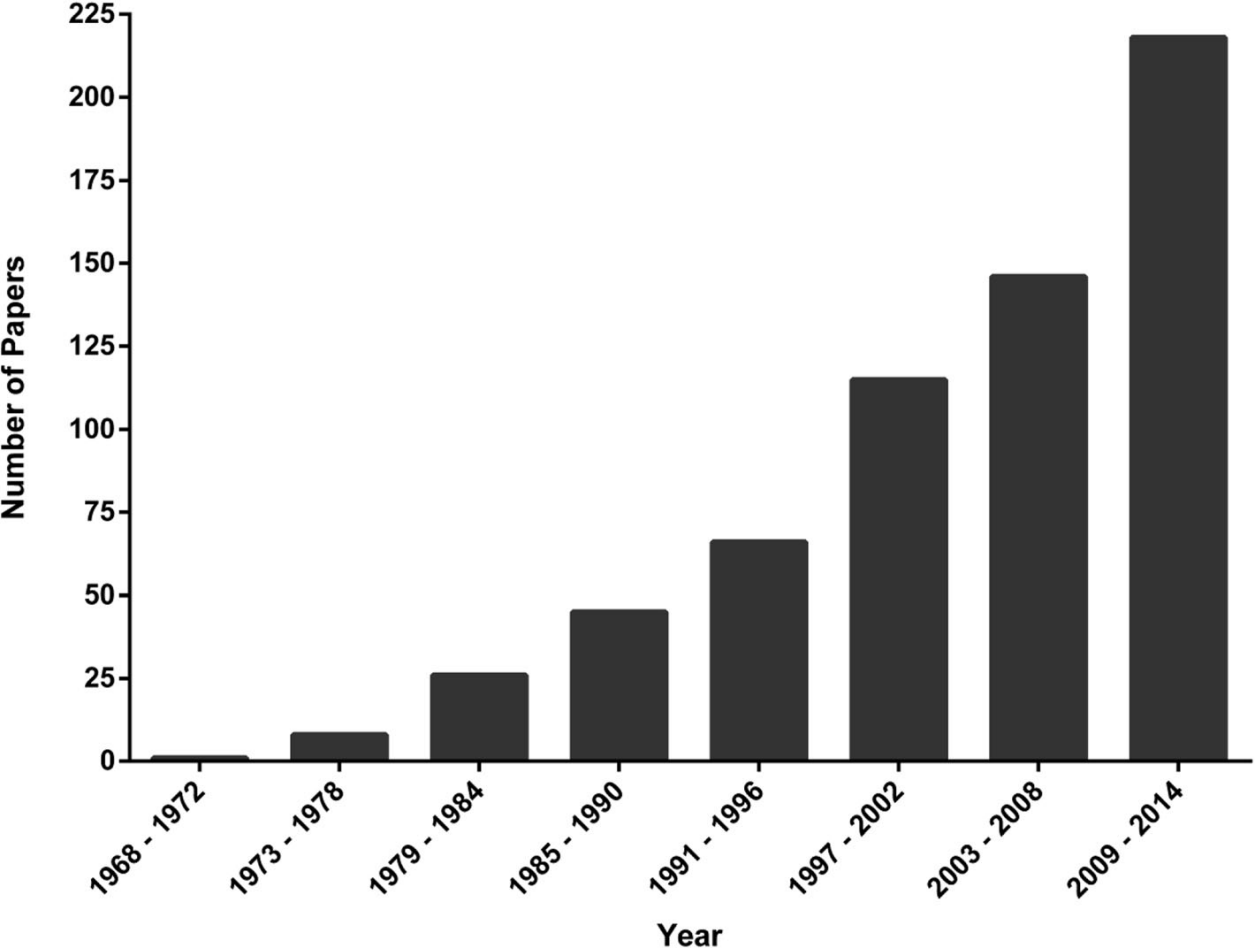
Distribution of papers across publication year (bin size of 5 years)

The numbers of articles at each stage are shown in a PRISMA [30] flow chart in Fig. 1. Articles relevant to the definitional review were identified following title and abstract review. This resulted in 635 papers included in our archive. Ten papers were removed due to language (only English-language articles were included), or the inability to identify a full-text version of the article. This left a total of 625 studies (full list available in the Digital Supplement), spanning 47 years (1968–2014; Fig. 2), published in 155 journals, written by 544 unique first authors. Papers from the North America were dominant (Table 1), almost two thirds of papers reported original research (Table 1), and papers represented the entire HPE training continuum (Table 2). Although a total of 18 different Health Professions were represented in our archive, more than half of the articles (*n* = 335) were from medicine (Table 2).

**Table 1:** Geographic distribution and type of papers included in our review

	**n**	**% of 625**
Location:		
North America	443	70.9
Europe	103	16.5
Oceania	37	5.9
Asia	21	3.4
South America	5	0.8
More than one location	16	2.6
Paper Type:		
Original research paper	403	64.5
Commentary/editorial	77	12.3
Review paper	70	11.2
Thesis	53	8.5
Conference paper or proceedings	2	0.3
Other:	20	3.2
-Innovation Report	19	3.0
-Brief report	1	0.2

**Table 2:** Representation of Health Professions and Level of Learner

**Health Professions**	**n**	**% of 625**
Medicine	335	53.6
Nursing	192	30.7
Dentistry	26	4.2
Physical Therapy	18	2.9
Occupational Therapy	16	2.6
Midwifery	6	1.0
Pharmacy	6	1.0
Paramedic	3	0.5
Dental Hygiene	2	0.3
Veterinary Medicine	2	0.3
Speech Language Pathology	1	0.2
Clinical Psychology	1	0.2
Diagnostic Imaging	1	0.2
Dietetics	1	0.2
Laboratory Technician	1	0.2
Respiratory Therapy	1	0.2
Chiropractic	1	0.2
Military Trauma Practice	1	0.2
Non-medical (e.g. psychology students)	12	1.9
**Level of Learner**		
Undergraduate	265	42.4
Postgraduate	130	20.8
Practicing Health Professional	193	30.9
Undefined	25	4.0
Non-Medical Populations	1	0.2

### Clinical reasoning terminology

Of the 625 papers included in this study, 230 papers (36.8%) used the verbatim term ‘clinical reasoning’ within the article. We used descriptive analyses to explore the relative proportion of papers that used the term clinical reasoning across the most frequently represented Health Professions in our database (medicine, nursing, dentistry, physical therapy and occupational therapy). Thirty-eight percent of papers in medicine used the term clinical reasoning (126/335), 27% in nursing (51/192), 23% in dentistry (6/26), 83% in physical therapy (15/18), and 81% in occupational therapy (13/16).

In the entire corpus of 625 papers, coders identified a total of 110 different terms used in reference to clinical reasoning. A total of six overarching categories of terminology were identified:

1. *reasoning skills* referred to the abilities needed in order to reason clinically—terms such as clinical skills, cognitive skills,
2. *reasoning performance* referred to aspirational goals for clinical reasoning to be attained—terms such as competency, acumen, or expertise,
3. *reasoning process* focused on the ‘how’ of clinical reasoning—proposing component processes or means by which the reasoning process unfolds (e.g. analytic reasoning, intuition, heuristics),
4. *outcome of reasoning* focused on the ‘what’ results from a reasoning process (e.g. a diagnosis, a management plan), the quality of that outcome (e.g. accuracy, quality), and the errors or failures in reasoning (e.g. bias, error),
5. *context of reasoning* included notions of ‘where’ the reasoning process is occurring ‘outside’ of the individual clinicians’ cognition, or factors that could influence that reasoning—including notions such as participatory approaches or shared decision making, or situational awareness which includes notions of influences on cognition that are more situationally or contextually derived,
6. *purpose/goal of reasoning* focused on the ‘why’ of clinical reasoning—for patient management, to determine a treatment, or to propose a diagnosis. A full list of terms for which consensus was reached and their categorization can be found in Table 3.

**Table 3:** Terms used to refer to clinical reasoning and their associated categorization

**Category**	**Smaller/Nested Code**	**Terms Identified in this Review:**	
Reasoning Skills		Clinical Skills	Critical Thinking
		Cognitive Skill	Reasoning
		Critical Reasoning	Reasoning Skills
Reasoning Performance	Expert Reasoning	Adaptive Expertise	Expert Reasoning
		Cognitive Expertise	Expertise
		Diagnostic Expertise	Medical Expertise
	Reasoning Competence	Clinical Competence	Diagnostic Acumen
		Clinical Performance	Diagnostic Performance
		Competency	
Reasoning Processes (Components)	Cognitive	Analytic Reasoning	Inductive and Deductive Reasoning
		Analytical Thinking	Intuition
		Backward Forward Reasoning	Intuitive Reasoning
		Backward Reasoning	Medical Information Processing
		Bayesian Probabilistic Thinking	Pattern Matching
		Cognitive Processes	Pattern Recognition
		Heuristics	‘Street Diagnosis’ Or ‘In The Blink Of The Eye’
		Hypothetico-Deductive Reasoning	
	Metacognitive	Metacognition	Self-Monitoring
		Reflective Thinking Skills	
Outcome Of Reasoning	Errors/Failures of Reasoning	Cognitive Bias	Medical Error
		Error Prevention	Premature Closure
		Judgement Errors	Reasoning Errors
	Outcome/Aim	Choice Of Treatment	Differential Diagnosis
		Classification	Management Plan
		Clinical Management Decisions	
		Diagnosis	
	Quality Of Outcome	Accuracy	Diagnostic Success
		Diagnostic Accuracy	
		Diagnostic And Management Quality	
Context Of Reasoning		Dialectical Reasoning	Shared Understanding
		Informed Decision Making	Situation Awareness
		Participatory Decision Making	Situational Judgement
		Shared Decision Making	
Purpose/Goal Of Reasoning	Different Goals Of Reasoning	Diagnostic Justification	
	Outcome-Focused Goal Of Reasoning	Case Management	Patient Management
		Diagnostic Reasoning	Therapeutic Reasoning
		Diagnostic Thinking	Treatment Decision Making

**Fig. 3:**
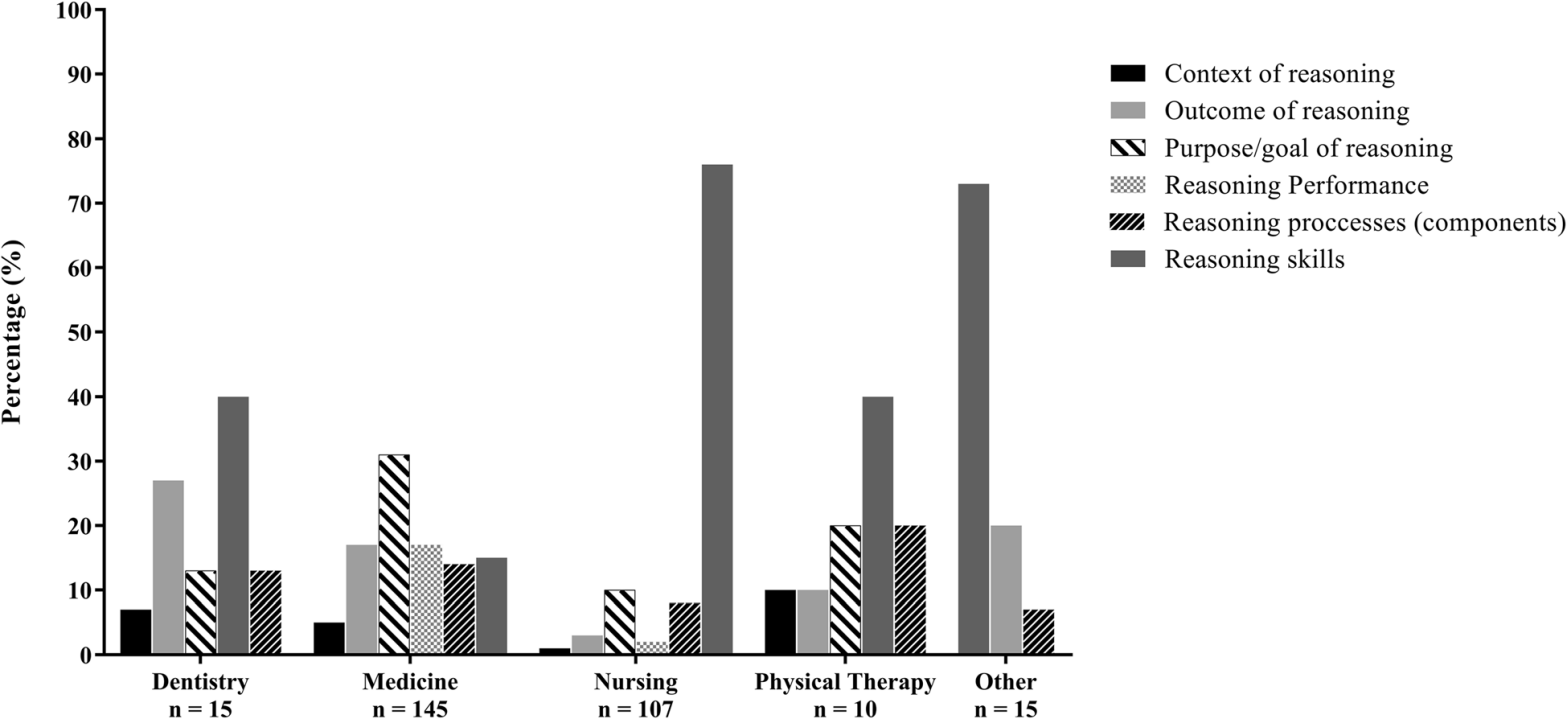
Presence of different categories of terminology for clinical reasoning across publications in various Health Professions

**Fig. 4:**
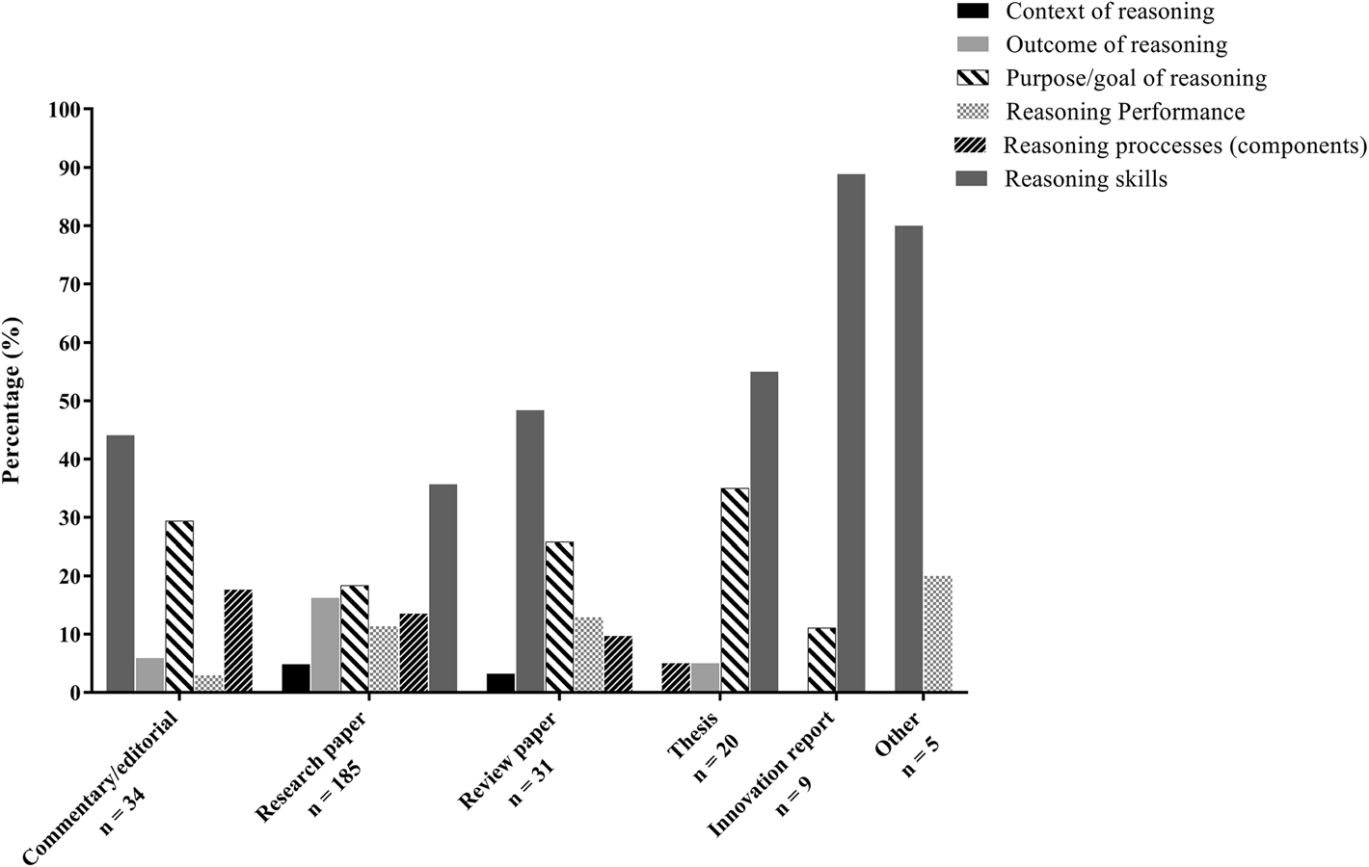
Presence of different categories of terminology for clinical reasoning across publication types

Categories of terms were differentially represented across Health Professions (Fig. 3). *Reasoning skills* descriptions dominated in dentistry, nursing, and physical therapy, whereas medicine had a high prevalence of terminology reflecting the *purpose or goal* of reasoning. When examining the presence of different categories of terms across publication type (Fig. 4), terminology reflecting *reasoning skills* was dominant in innovation reports, theses, and review papers, whereas *skills* and *purpose or goal* of reasoning terminology were relatively balanced in original research papers and commentaries or editorials.

**Fig. 5:**
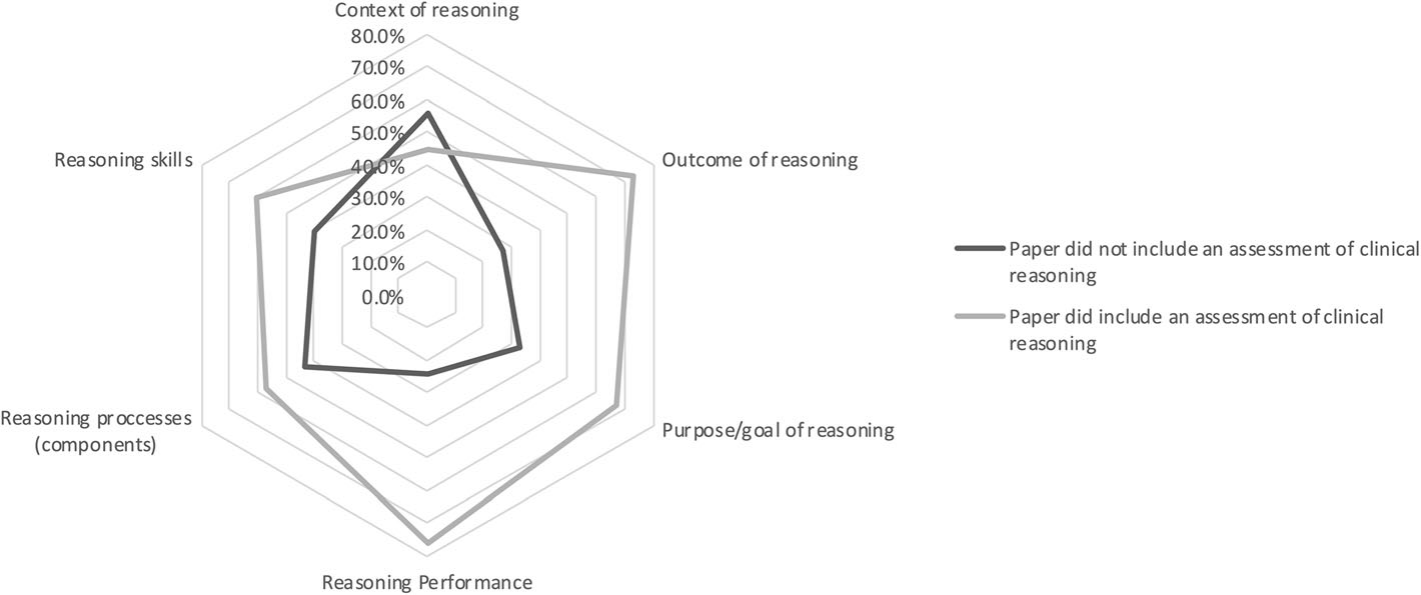
Presence of each category of terminology in papers that report on an assessment of clinical reasoning, compared to those that do not report on an assessment of clinical reasoning

We explored how different categories of terms related to the likelihood that a given work included an assessment of clinical reasoning compared to those that did not (Fig. 5). Papers reporting on assessments were much more likely to describe clinical reasoning in terms of *reasoning performance, purpose/goal of reasoning,* and *outcome of reasoning,* and less likely to use terminology reflecting the *context of reasoning* than other categories of terminology.

## Discussion

This review explored how clinical reasoning is represented within the Health Professions Education (HPE) literature. Through this review, a group of scholars from different professions, different training backgrounds, and different perspectives on clinical reasoning [28], engaged in a synthesis to explore how clinical reasoning is described in the HPE literature. We analyzed papers spanning nearly half a century, representing 18 different Health Professions, levels of learners across the continuum, and a variety of publication types. We do not claim that these papers represent the entire corpus of writing on the topic of clinical reasoning in HPE; we argue instead that it represents a broad sampling of literature informing this topic and creates a foundation to map different areas of focused attention, and perhaps differing conceptualizations of clinical reasoning.

Just over one third of articles in this review contained the verbatim term ‘clinical reasoning’ within the title, abstract, or body of the article. Articles from the fields of physical and occupational therapy were the most likely to include the exact phrase ‘clinical reasoning’; this may be due, in part, to the presence of very explicit frameworks and definitions of clinical reasoning within these rehabilitation professions [2, 8, 31–34]. For example, reasoning is understood as a cognitive or metacognitive process that guides clinical practice and includes: procedural, interactive, conditional, narrative, and pragmatic reasoning [33]. These explicit descriptions of clinical reasoning likely support relative uniformity in the conceptual framework underlying the term in these professions.

In lieu of the term ‘clinical reasoning’, we identified terms referring to clinical reasoning, grouped into six overarching categories, which appear to represent different dimensions of focus in the operationalization of clinical reasoning. More specifically, each category appears to focus on different aspects or components of clinical reasoning, with terms variously focused on the ‘why’, the ‘how’, the ‘where’, the ‘what’ or the ‘what should’ result from a reasoning process. These six categories of terms were not used uniformly across the Health Professions. Articles from medicine (dominant in our database), tended to use language associated with the *purpose or goal of reasoning* (e.g. diagnostic reasoning), whereas articles reporting on clinical reasoning in nursing tended to use language reflecting reasoning as a *skill* (e.g. critical thinking). These categories of terminology appear to prioritize different components or aspects of clinical reasoning—perhaps suggesting different conceptualizations, understandings, or operationalizations [35] of what constitutes clinical reasoning across the Health Professions.

When examining across publication type, we saw a relatively consistent presence of language reflecting the *purpose or goal of reasoning*, or *reasoning as a skill*, with the exception of innovation reports where the language of *reasoning as a skill* dominated. This finding may indicate that in educational innovations—publications describing new approaches to teaching and learning—reasoning may be expressed as a teachable or learnable skill rather than a process or contextually-bound experience.

Finally, we examined the presence of these six categories of terminology across papers that did, or did not, include the description of an assessment. The only category of terminology less likely to be present in a paper reporting on assessment of clinical reasoning was language around the *context of reasoning* (e.g. participatory decision-making). This finding may suggest that either this category of language has not been broadly adopted by the assessment literature or this conceptualization of clinical reasoning may be more difficult to assess and perhaps less amenable to assessment approaches.

To summarize our findings, the literature included in this synthesis is broad and represents many different facets of the HPE literature on clinical reasoning. Further, there are a multitude of terms being used to refer to clinical reasoning. However, based on their differential representation across paper type, health profession, and the inclusion of an assessment, these terms do not appear to be used synonymously. This result suggests that clinical reasoning may be an overarching concept, rather than a singularly definable entity in itself [35]. Rather, the concept of clinical reasoning appears to manifest, be operationalized, or crystalized differently depending on the context—whether across individual health professions, different publication types, or assessment focus.

The purpose of this review was to provide a concrete description of the variability within the concept of clinical reasoning [36], respecting the differences across Health Professions, without creating a hierarchy of terminology, operationalizations, or conceptualizations of a concept, nor homogenizing our findings into one universal definition of clinical reasoning across the Health Professions. Our purpose was to map the breadth of literature, and to attempt to provide an organizational framework for various understandings of clinical reasoning. While clinical reasoning has been referred to as a multi-dimensional construct [9, 10], the likely presence of multiple conceptualizations of clinical reasoning, suggested by the different terms used to label it, has important implications for teaching, assessment, and research within and across the Health Professions. One could imagine that an assessment based on a conceptualization of clinical reasoning as a contextually-bound experiential phenomenon may focus on very different dimensions of reasoning than one based on a conceptualization focused on the outcome of reasoning. Similarly, educational programs or interventions would likely take very different shapes if one were to focus on reasoning as a skill (i.e. focus on transferable approaches to reasoning) as opposed to focusing on the purpose or goal of reasoning (e.g. focus on the justification of a diagnosis). Summarizing particular approaches to teaching or assessment that reflect these different conceptualizations of reasoning are beyond the scope of the current review, but remain an important avenue for future research.

This study has limitations. We acknowledge that the corpus of studies included in this review does not represent the full literature available on the topic of clinical reasoning, and the distribution of terminologies, use of the term ‘clinical reasoning’, and distribution of studies may not generalize to the entire literature available on clinical reasoning and related concepts. However, we believe that the breadth represented in this review allows for an initial mapping of some of the different contexts, terms, and perhaps conceptualizations of clinical reasoning present in the HPE literature. While members of our team represent a variety of expertise and experiences, our team did not include nursing as an area of expertise. Given the representation of articles from nursing within our database, that particular perspective may have been beneficial to our analytical team. Future work should include representation from a broader range of health professionals in order to better situate clinical reasoning as a potential area for interprofessional or team-based [37] education.

Several areas for consideration and educational development remain. With ‘competence’ as a final goal, explicit identification of a (or perhaps several) conceptualization(s) of clinical reasoning is required in order to describe and develop performance profiles of trainees. Further, these different dominant conceptualizations of clinical reasoning across the Health Professions will - and likely already do - inform the complex context of both Interprofessional Education (IPE) and Interprofessional Collaborative Practice. IPE competencies currently do not explicitly focus on clinical reasoning [38], yet clinical reasoning has been identified as important across Health Professions and thus may be reasonable, or even essential, for IPE to address. However, as terminology or perhaps even conceptualizations of clinical reasoning differ across Health Professions, this may prove challenging as different professions’ educational programs may reflect different understandings, operationalizations, or prioritization of different areas of focus of this multifaceted concept. Our work provides an initial structure to begin to address this complex educational and practice challenge, without proposing an interprofessional unified definition of clinical reasoning relevant to all Health Professions.

## Conclusion

The variability in terminology used to describe clinical reasoning across the Health Professions Education literature may lead to unclear communication within the clinical reasoning community, and perhaps difficulty in operationalizing the concept of clinical reasoning for teaching and assessment in the Health Professions. We encourage those involved in the study, teaching, and assessment of clinical reasoning to carefully consider and make explicit their intended understanding of clinical reasoning in order to support better communication, teaching, and assessment of clinical reasoning.

## Supplementary information

**Additional file 1.** Search strategy used for this review included search terms for three constructs: clinical reasoning, assessment, and education.

## Data Availability

All data and materials are available from the authors upon request. Data collection materials are included in the Appendix, and all other data is drawn from published manuscripts.

## Abbreviation

HPE: Health Professions Education
