# Supplementary material for: Mapping clinical reasoning literature across the health professions: a scoping review
Source: BMC Med Educ. 2020 Apr 7;20:107. doi: 10.1186/s12909-020-02012-9 (PMC7140328; doi:10.1186/s12909-020-02012-9)
Supplement: Supplementary file 1 — Additional file 1. Search strategy used for this review included search terms for three constructs: clinical reasoning, assessment, and education. [file 12909_2020_2012_MOESM1_ESM.docx]

Appendix 1:

Search strategy included search terms for three constructs: clinical reasoning, assessment, and education.

Subheadings and terms for ‘reasoning’

1. Clinical Competence +
2. Education  +
3. exp Education, Professional +
4. Educational Measurement +
5. Evaluation Studies +
6. Mentors +
7. Preceptorship +

Subheadings and terms for ‘assessment’

1. Clinical Competence +
2. Education  +
3. exp Education, Professional +
4. Educational Measurement +
5. Evaluation Studies +
6. Mentors +
7. Preceptorship +

Subheadings and terms for ‘education’

1. Competency Based Education
2. Curriculum
3. Education
4. exp Education, Professional [includes Internship & Residency; Clinical Clerkship; Education, Medical]
5. exp In-service Training [includes Staff Development]
6. exp Learning [may select a few terms]
7. exp Students, Health Occupations [change from Students in set 55]

exp Teaching [includes Problem Based Learning]

Appendix 2: Extraction form used for the current study

1. Citation type:
   1. Original research paper
   2. Review Paper
   3. Commentary/editorial
   4. Conference paper or proceedings
   5. Book
   6. Thesis
   7. Other (please list)

1. Please indicate what type of review the authors report conducting
   1. Systemic
   2. Systemic with meta-analysis
   3. Structured
   4. Narrative
   5. Realist
   6. Scoping
   7. Other (please indicate)
   8. Not stated, but I infer the type of review was (please indicate)

1. Geographic location of the study. If location of the study (i.e. study population) is not possible, or not relevant, select the location of the majority of the authors.
   1. North America
   2. South America
   3. Europe
   4. Asia
   5. Europe
   6. Oceania
   7. More than one location (please list)
2. This is about the focal population in the article. For primary research articles this would include participants. If it is a review article, it may focus on a particular health profession. Choose all that apply (all health professionals and all levels)
   1. Medicine
      1. Level of learner (medicine)
         1. Pre-medical (including admissions)
         2. Undergraduate
            1. Pre-clinical
            2. Clinical
         3. Postgraduate
            1. Resident or House Officer
            2. Fellow
         4. Practicing physician/CPD
         5. Other
   2. Nursing
      1. Level of learner (Nursing)
         1. Pre-nursing (including admissions)
         2. Undergraduate
            1. Pre-clinical
            2. Clinical
         3. Postgraduate
         4. Practicing nurses/CHPD
         5. Other
   3. Dentistry
      1. Level of learner (Dentistry)
         1. Pre-dentistry (including admissions)
         2. Pre-clinical
         3. Clinical
         4. Postgraduate
         5. Practicing nurses/CHPD
         6. Other
   4. Physical Therapy
      1. Level of learner (Physical Therapy)
         1. Pre-PT (including admissions)
         2. BSc
         3. Masters Entry-level
         4. Clinical Doctorate in PT
         5. Pre-Clinical
         6. Clinical
         7. Intern
         8. Other
   5. Occupational Therapy
      1. Level of learner (Occupational Therapy)
         1. Pre-OT (including admissions)
         2. BSc
         3. Masters Entry-level
         4. Clinical Doctorate in OT
         5. Pre-Clinical
         6. Clinical
         7. Intern
         8. Other
   6. Speech Language Pathology
      1. Level of learner (Speech Language Pathology)
         1. Pre-SLP (including admissions)
         2. Pre-Clinical
         3. Clinical
         4. Masters Level
         5. Doctoral Level
         6. Other
   7. Physician Assistants
      1. Level of learner (Physician Assistants)
         1. Pre-PA (including admissions)
         2. Pre-Clinical
         3. Clinical
         4. Graduate level
         5. Other
   8. Osteopathy
      1. Level of learner (Osteopathy)
         1. Pre-osteopathy (including admissions)
         2. Pre-Clinical
         3. Clinical
         4. Graduate level
         5. Other
   9. Nutritionist
      1. Level of learner (Nutritionist)
         1. Pre-nutritionist (including admissions)
         2. Pre-Clinical
         3. Clinical
         4. Graduate level
         5. Other
   10. Chiropractic
       1. Level of learner (Chiropractic)
          1. Pre-chiropractic (including admissions)
          2. Pre-Clinical
          3. Clinical/Intern
          4. Masters level
          5. Doctorate of Chiropractic
          6. Other
   11. Other (please indicate profession and level)
3. Is this paper presenting data from an original research study?
   1. Yes
   2. No

1. Please summarize the main purpose of the study (using your own words, or quoting from the article. If you are quoting, please use quotation marks and indicate the page you are quoting from).
2. The study conducted was (please choose all that apply)
   1. Experimental
   2. Observational
   3. Descriptive
   4. Quasi-experimental
   5. Mixed methods
   6. Other (please describe)

1. Approach to data collection was (please check all that apply):
   1. Qualitative (Please summarize what kind of data was collected e.g. field notes of observations, interviews, focus groups, etc.)
   2. Quantitative (Please summarize what kind of data was collected e.g. diagnostic accuracy, evaluation of management plan, etc.)
   3. Mixed methods (Please summarize what kind of data was collected in each study arm)
   4. Other (Please describe)
2. The setting of the study was (please check all that apply and provide a brief description):
   1. Clinical setting (please provide a brief description)
   2. Lab-based experimental setting (please provide a brief description)
   3. Simulation setting (please provide a brief description)
   4. Virtual setting/ online patient cases (please provide a brief description)
   5. Other (please describe)
3. Does this paper report on an assessment of CR?
   1. Yes
   2. No
   3. Comments regarding assessment of CR (optional):
4. Is the term 'clinical reasoning' used in this paper?
   1. Yes
   2. No
5. "Is an explicit definition of clinical reasoning included? An example could be: “For the purpose of this study, we define clinical reasoning as…”
   1. Yes
   2. No
   3. Please copy and paste definition(s) of clinical reasoning present in this paper (please use quotation marks, and indicate page numbers for source quotes as appropriate).
6. Is an implicit definition of clinical reasoning included? (i.e. is a definition implied/insinuated based on the measures of clinical reasoning or the manner the study/paper describes clinical reasoning?). An example could be: “We assessed subjects’ ability to efficiently and effectively come to a diagnostic conclusion…” This is very dependent on your ‘feel’ of the paper.
   1. Yes
   2. No
   3. What is the definition? If quotes are helpful here, please include quotation marks and page numbers. Otherwise please provide your impressions.
7. Is a term other than clinical reasoning used in this article? Examples might include ‘clinical problem solving’, ‘diagnostic reasoning’, etc.? You will be able to enter up to three different terms, but please only enter one at a time (you will be asked to provide the term, then the implicit or explicit definition for that term prior to moving to the second term).
   1. Yes
   2. No
   3. Please provide the term
8. Is an explicit definition of this term included? An example could be: “For the purpose of this study, we defined ‘clinical problem-solving’ as…”
   1. Yes
   2. No
   3. Please copy and paste definition(s) of the abovementioned word present in this paper (please use quotation marks, and indicate page numbers for source quotes as appropriate).
9. Is there an implicit definition of the above-mentioned term? (i.e. is it implied/insinuated based on the measures of clinical reasoning or the manner the study/paper describes clinical reasoning?). An example could be: “We assessed subjects’ ability to efficiently and effectively come to a diagnostic conclusion…” This is very dependent on your ‘feel’ of the paper.
   1. Yes
   2. No
   3. What is the definition? If quotes are helpful here, please include quotation marks and page numbers, otherwise please provide your impressions.

*Note: items 16 through 18 were repeated to a maximum of three times.*

1. Is an explicit theoretical framework relating to clinical reasoning included in this paper (e.g. a certain theory, model, or approach referred to or cited?) Examples could be: “In this study, we used an information processing approach to...” or “Using situated cognition as a theoretical framework, we investigated…”
   1. Yes
   2. No
   3. Please provide the framework, theory, model or approach.
   4. Please copy the citation provided when mentioning the framework, theory, model or approaches (if none is provided, also please indicate).
2. Is an implicit theoretical framework relating to clinical reasoning included in this paper? An example might be: “students need organized knowledge, so we assessed this using SCT” (implies script theory).
   1. Yes
   2. No
   3. If quotes are helpful here, please include quotation marks and page numbers related to the implicit theoretical framework. Otherwise please provide your impressions.
3. In your impression of this article, are the authors referring to clinical reasoning (or associated term) as (please check all that apply and please provide comments)
   1. A process
   2. An outcome
   3. A skill
   4. A behavior
   5. A task
   6. Other (please describe)
   7. I don't know
4. Based on your knowledge, is this paper appropriate for inclusion in the definitional review?
   1. Yes
   2. No
   3. Why not?
5. General comments:

Digital supplement: Complete list of works included in review.

Aaraas IJ, Holtedahl K, Anvik T, Bentzen N, Berg E, Fleten N, et al. Examination of final-year medical students in general practice. Scandinavian journal of primary health care. 2007;25(4):198.

Abbey LM. Interactive multimedia patient simulations in dental and continuing dental education. Dental clinics of North America. 2002;46(3):575.

Abboudi H, Ahmed K, Normahani P, Abboudi M, Kirby R, Challacombe B, et al. Decision making in urological surgery. International Urology & Nephrology. 2012;44(3):701.

Abdelghany A, Nolan A, Freeman R. Treating patients with dry mouth: general dental practitioners' knowledge, attitudes and clinical management. British dental journal. 2011;211(10):E21.

Abendroth M, Graven LJ. Integrating care of older adults into the nursing curriculum: a case exemplar project. Journal of Nursing Education. 2013;52(9):529.

Abendroth M, Harendza S, Riemer M. Clinical decision making: a pilot e-learning study. The clinical teacher. 2013;10(1):51.

Abraham RR, Upadhya S, Torke S, Ramnarayan K. Clinically oriented physiology teaching: strategy for developing critical-thinking skills in undergraduate medical students. Advances in Physiology Education. 2004;28(1-4):102.

Adams H, Wills R, Shannon F, Hadac R, Weinstein P. Assessing clinical judgment in dentistry: practitioner evaluation of patient management problems. Journal of the American College of Dentists. 1979;46(3):187.

Adams MH, Whitlow JF, Stover LM, Johnson KW. Critical thinking as an educational outcome: an evaluation of current tools of measurement. Nurse educator. 1996;21(3):23.

Adamson KA, Gubrud P, Sideras S, Lasater K. Assessing the reliability, validity, and use of the Lasater Clinical Judgment Rubric: three approaches. Journal of Nursing Education. 2012;51(2):66.

Adamson KA, Kardong-Edgren S. A method and resources for assessing the reliability of simulation evaluation instruments. Nursing Education Perspectives. 2012;33(5):334.

Adrales GL, Chu UB, Witzke DB, Donnelly MB, Hoskins D, Mastrangelo MJ, et al. Evaluating minimally invasive surgery training using low-cost mechanical simulations. Surgical endoscopy. 2003;17(4):580.

Adrales GL, Park AE, Chu UB, Witzke DB, Donnelly MB, Hoskins JD, et al. A valid method of laparoscopic simulation training and competence assessment. Journal of Surgical Research. 2003;114(2):156.

Agbedia CO, Ofi B, Ibeagha JE. Causal model of clinical judgment of practising nurses, in selected hospitals in Delta State, Nigeria. West African Journal of Nursing. 2008;19(2):111.

Agha H, Arora S, Sevdalis N. Quantitative assessment of expert and novice surgeons' thinking processes: an application to hernia repair. American Journal of Surgery. 2011;202(1):110.

Ahern DE. Diagnostic imaging, decision making, and the Internet: opportunity or threat? Seminars in Radiologic Technology. 1999;7(4):128.

Ahluwalia S, Swanwick T. Specialty training for general practice in the United Kingdom. Journal of Ambulatory Care Management. 2008;31(3):276.

Ahmed H, Rhydderch M, Matthews P. Can knowledge tests and situational judgement tests predict selection centre performance? Medical education. 2012;46(8):777.

Ahmed H, Rhydderch M, Matthews P. Do general practice selection scores predict success at MRCGP? An exploratory study. Education for Primary Care. 2012;23(2):95.

Ajjawi R, Higgs J. Core components of communication of clinical reasoning: a qualitative study with experienced Australian physiotherapists. Advances in Health Sciences Education. 2012;17(1):107.

Akinsanya C, Williams M. Concept mapping for meaningful learning. Nurse education today. 2004;24(1):41.

Albanese MA, Mitchell S. Problem-based learning: a review of literature on its outcomes and implementation issues. Academic Medicine. 1993;68(1):52.

Alexander MK, Giguere B. Critical thinking in clinical learning: a holistic perspective. Holistic nursing practice. 1996;10(3):15.

Ali J, Ahmed N, Jacobs LM, Luk SS. The Advanced Trauma Operative Management course in a Canadian residency program. Canadian Journal of Surgery. 2008;51(3):185.

Ali NS, Bantz D, Siktberg L. Validation of critical thinking skills in online responses. Journal of Nursing Education. 2005;44(2):90.

Alien P, Lauchner K, Bridges RA, Francis-Johnson P, McBride SG, Olivarez A. Evaluating continuing competency: a challenge for nursing. Journal of continuing education in nursing. 2008;39(2):81.

Alireza M, Ehsan Rajabi R, Shokoufeh S, Remy MJPR. How does patient management knowledge integrate into an illness script? Education for Health: Change in Learning & Practice. 2012;25(3):153.

Allareddy V, Havens AM, Howell TH, Karimbux NY. Evaluation of a new assessment tool in problem-based learning tutorials in dental education. Journal of dental education. 2011;75(5):665.

Allen GD, Rubenfeld MG, Scheffer BK. Reliability of assessment of critical thinking. Journal of Professional Nursing. 2004;20(1):15.

Allen KL, More FG. Clinical simulation and foundation skills: an integrated multidisciplinary approach to teaching. Journal of dental education. 2004;68(4):468.

Allen VG, Arocha JF, Patel VL. Evaluating evidence against diagnostic hypotheses in clinical decision making by students, residents and physicians. International journal of medical informatics. 1998;51(2-3):91.

Al-Naami MY, El-Tinay OF, Khairy GA, Mofti SS, Anjum MN. Improvement of psychometric properties of the objective structured clinical examination when assessing problem solving skills of surgical clerkship. Saudi medical journal. 2011;32(3):300.

Alsharif NZ. Medicinal chemistry and therapeutic relevance of angiotensin-converting enzyme inhibitors. American Journal of Pharmaceutical Education. 2007;71(6):123.

Altun I. The perceived problem solving ability and values of student nurses and midwives. Nurse education today. 2003;23(8):575.

Anastakis DJ, Cohen R, Reznick RK. The structured oral examination as a method for assessing surgical residents. American Journal of Surgery. 1991;162(1):67.

Andersen DK. How can educators use simulation applications to teach and assess surgical judgment? Academic Medicine. 2012;87(7):934.

Andersen SM, Harthorn BH. The Diagnostic Knowledge Inventory: a measure of knowledge about psychiatric diagnosis. Journal of clinical psychology. 1989;45(6):999.

Anderson AS, Botticelli MG. Evaluating M.D.-level competence in internal medicine. Journal of medical education. 1981;56(7):587.

Anderson C, Sullivan JP, Flynn-Evans EE, Cade BE, Czeisler CA, Lockley SW. Deterioration of neurobehavioral performance in resident physicians during repeated exposure to extended duration work shifts. Sleep. 2012;35(8):1137.

Anderson JD, Jay SJ, Weng HC, Anderson MM. Studying the effect of clinical uncertainty on physicians' decision-making using ILIAD. Medinfo. 1995;8(Pt 2):869.

Andrew RB, Alan LH. The Effect of Nonlinear Transformations on a Likert Scale. Evaluation and the Health Professions. 1983:483.

Andrews M, Jones PR. Problem-based learning in an undergraduate nursing programme: a case study. Journal of advanced nursing. 1996;23(2):357.

Angel BF, Duffey M, Belyea M. An evidence-based project for evaluating strategies to improve knowledge acquisition and critical-thinking performance in nursing students. Journal of Nursing Education. 2000;39(5):219.

Anique De B, Gino C, Jeroen Van M. Available but irrelevant: when and why information from memory hinders diagnostic reasoning. Medical education. 2010;44(10):948.

Annie C, Mélanie L, Denis B, Johanne D. Community occupational therapists. Australian Occupational Therapy Journal. 2010;57(6):356.

Anthony EV. Premature Conclusions in Diagnostic Reasoning. Journal of medical education. 1985;60(4):302.

Aquilino ML. Epistemological beliefs, clinical knowledge and professional experience related to problem perception in nursing diagnosis. 1993:122 p.

Arbesman M, Scheer J, Lieberman D. Using AOTA's critically appraised topic (CAT) and critically appraised paper (CAP) series to link evidence to practice: supporting the integration of research evidence, practitioner expertise, and client perspectives in the clinical decision-making process. OT Practice. 2008;13(5):18.

Ark TK, Brooks LR, Eva KW. Giving learners the best of both worlds: do clinical teachers need to guard against teaching pattern recognition to novices? Academic Medicine. 2006;81(4):405.

Ark TK, Brooks LR, Eva KW. The benefits of flexibility: the pedagogical value of instructions to adopt multifaceted diagnostic reasoning strategies. Medical education. 2007;41(3):281.

Arslanian-Engoren C. Patient cues that predict nurses' triage decisions for acute coronary syndromes. Applied Nursing Research. 2005;18(2):82.

Arslanian-Engoren C. Explicating nurses' cardiac triage decisions. Journal of Cardiovascular Nursing. 2009;24(1):50.

Arthur SE. What goes around comes around: Return of the hypothetico-deductive strategy. Teaching and learning in medicine. 1994;6(2):121.

Arthur SE. Thinking about Diagnostic Thinking: A 30-Year Perspective. Advances in Health Sciences Education. 2009;14.

Ashcraft ALS. The clinical reasoning of expert acute care registered nurses in pre-cardiopulmonary arrest events. 2001:424 p.

Axelsson L, Bjorvell C, Mattiasson AC, Randers I. Swedish Registered Nurses' incentives to use nursing diagnoses in clinical practice. Journal of Clinical Nursing. 2006;15(8):936.

Ayres-de-Campos D, Bernardes J, Costa-Pereira A, Pereira-Leite L. Inconsistencies in classification by experts of cardiotocograms and subsequent clinical decision. British Journal of Obstetrics & Gynaecology. 1999;106(12):1307.

Azer SA. Use of portfolios by medical students: significance of critical thinking. Kaohsiung Journal of Medical Sciences. 2008;24(7):361.

Babbott D, Halter WD. Clinical problem-solving skills of internists trained in the problem-oriented system. Journal of medical education. 1983;58(12):947.

Babyar SR, Rosen E, Sliwinski MM, Krasilovsky G, Holland T, Lipovac M. Physical therapy students' self-reports of development of clinical reasoning: a preliminary study. Journal of allied health. 2003;32(4):227.

Bader JD, Shugars DA. Variation in dentists' clinical decisions. Journal of public health dentistry. 1995;55(3):181.

Badger LW, deGruy F, Hartman J, Plant MA, Leeper J, Ficken R, et al. Stability of standardized patients' performance in a study of clinical decision making. Family medicine. 1995;27(2):126.

Badger MJ, Lookinland S, Tiedeman M, Anderson V, Eggett D. Nurse practitioners' treatment of febrile infants in Utah: comparison to physician practice nationally. Journal of the American Academy of Nurse Practitioners. 2002;14(12):540.

Bailey RH, Aron DC. The diagnostic dilemma of incidentalomas. Working through uncertainty. Endocrinology & Metabolism Clinics of North America. 2000;29(1):91.

Bajgier J, Bender J, Ries R. Use of templates for clinical documentation in psychiatric evaluations-beneficial or counterproductive for residents in training? International journal of psychiatry in medicine. 2012;43(1):99.

Bakalis N. Clinical decision-making in cardiac nursing: a review of the literature. Nursing Standard. 2006;21(12):39.

Bakalis NA. A European perspective of clinical decision-making. CONNECT: The World of Critical Care Nursing. 2007;5(3):66.

Baker CR. Reflective learning: a teaching strategy for critical thinking. Journal of Nursing Education. 1996;35(1):19.

Baker DC. Nursing reasoning model. Nurse educator. 2001;26(5):203.

Baker EA, Connell KJ, Bordage G, Sinacore J. Can diagnostic semantic competence be assessed from the medical record? Academic Medicine. 1999;74(10 Suppl):S13.

Bakken LL. Role of experience and context in learning to diagnose Lyme disease. Journal of Continuing Education in the Health Professions. 2002;22(3):131.

Balla JI, Biggs JB, Gibson M, Chang AM. The application of basic science concepts to clinical problem-solving. Medical education. 1990;24(2):137.

Balslev T, Jarodzka H, Holmqvist K, Grave Wd, Muijtjens AM, Eika B, et al. Visual expertise in paediatric neurology. European Journal of Paediatric Neurology. 2012;16(2):161.

Barrett JF, Jarvis GJ, Macdonald HN, Buchan PC, Tyrrell SN, Lilford RJ. Inconsistencies in clinical decisions in obstetrics. Lancet. 1990;336(8714):549.

Barrows HS, Feltovich PJ. The clinical reasoning process. Medical education. 1987;21(2):86.

Barrows HS, Norman GR, Neufeld VR, Feightner JW. The clinical reasoning of randomly selected physicians in general medical practice. Clinical & Investigative Medicine - Medecine Clinique et Experimentale. 1982;5(1):49.

Bartlett DJ, Cox PD. Measuring change in students' critical thinking ability: implications for health care education. Journal of allied health. 2002;31(2):64.

Bartlett R, Bland A, Rossen E, Kautz D, Benfield S, Carnevale T. Evaluation of the Outcome-Present State Test Model as a way to teach clinical reasoning. Journal of Nursing Education. 2008;47(8):337.

Bashook PG. A conceptual framework for measuring clinical problem-solving. Journal of medical education. 1976;51(2):109.

Beck AL, Bergman DA. Using structured medical information to improve students' problem-solving performance. Journal of medical education. 1986;61(9 Pt 1):749.

Becker HA, MacCabe N. Indicators of critical thinking, communication, and therapeutic intervention among first-line nursing supervisors. Nurse educator. 1994;19(2):15.

Beckie TM, Lowry LW, Barnett S. Assessing critical thinking in baccalaureate nursing students: a longitudinal study. Holistic nursing practice. 2001;15(3):18.

Beckstead JW, Stamp KD. Understanding how nurse practitioners estimate patients' risk for coronary heart disease: a judgment analysis. Journal of advanced nursing. 2007;60(4):436.

Beeken JE. The relationship between critical thinking and self-concept in staff nurses and the influence of these characteristics on nursing practice. Journal of Nursing Staff Development. 1997;13(5):272.

Bell E, Horton G, Blashki G, Seidel BM. Climate change: could it help develop 'adaptive expertise'? Advances in Health Sciences Education. 2012;17(2):211.

Bell JG, Kanellitsas I, Shaffer L. Selection of obstetrics and gynecology residents on the basis of medical school performance. American Journal of Obstetrics & Gynecology. 2002;186(5):1091.

Belman S, Murphy J, Steiner JF, Kempe A. Consistency of triage decisions by call center nurses. Ambulatory Pediatrics. 2002;2(5):396.

Bereby-Meyer Y, Meyer J, Budescu DV. Decision making under internal uncertainty: the case of multiple-choice tests with different scoring rules. Acta Psychologica. 2003;112(2):207.

Bernard C, Jacques T, Henny PAB. Scripts and medical diagnostic knowledge: Theory and applications for clinical reasoning instruction and research. Academic Medicine. 2000;75(2):182.

Berner ES. Paradigms and problem-solving: a literature review. Journal of medical education. 1984;59(8):625.

Berner ES, Graber ML. Overconfidence as a Cause of Diagnostic Error in Medicine. American Journal of Medicine. 2008;121(5 SUPPL.):S2.

Berry P. Achieving independence: a decision-making framework for doctors in training. Clinical Medicine. 2008;8(5):512.

Bersky AK. The validity of a new test of nursing competence. 1994:143 p.

Bersky AK, Krawczak J, Kumar TD. Computerized Clinical Simulation Testing. A new look for the NCLEX-RN examination? Nurse educator. 1998;23(1):20.

Beverly H. Computer-based versus high-fidelity mannequin simulation in developing clinical judgment in nursing education. 2013:91 p.

Bhugra D, Malliaris Y, Gupta S. How shrinks think: decision making in psychiatry. Australasian Psychiatry. 2010;18(5):391.

Biese KJ, Roberts E, LaMantia M, Zamora Z, Shofer FS, Snyder G, et al. Effect of a geriatric curriculum on emergency medicine resident attitudes, knowledge, and decision-making. Academic Emergency Medicine. 2011;18(Suppl 2):S92.

Bissessur SW, Geijteman EC, Al-Dulaimy M, Teunissen PW, Richir MC, Arnold AE, et al. Therapeutic reasoning: from hiatus to hypothetical model. Journal of evaluation in clinical practice. 2009;15(6):985.

Black ME. Student nurses' clinical decision-making: key to professional practice. 1997:176 p.

Blissett S, Cavalcanti RB, Sibbald M. Should we teach using schemas? Evidence from a randomised trial. Medical education. 2012;46(8):815.

Blum JM, Kheterpal S, Tremper KK. A comparison of anesthesiology resident and faculty electronic evaluations before and after implementation of automated electronic reminders. Journal of clinical anesthesia. 2006;18(4):264.

Bojalil R, Guiscafre H, Espinosa P, Viniegra L, Martinez H, Palafox M, et al. A clinical training unit for diarrhoea and acute respiratory infections: an intervention for primary health care physicians in Mexico. Bulletin of the World Health Organization. 1999;77(11):936.

Bondy KN, Koenigseder LA, Ishee JH, Williams BG. Psychometric properties of the California Critical Thinking Tests. Journal of nursing measurement. 2001;9(3):309.

Bordage G. Elaborated knowledge: a key to successful diagnostic thinking. Academic Medicine. 1994;69(11):883.

Bordage G, Carretier H, Bertrand R, Page G. Comparing times and performances of French- and English-speaking candidates taking a national examination of clinical decision-making skills. Academic Medicine. 1995;70(5):359.

Borduas F, Gagnon R, Lacoursiere Y, Laprise R. The longitudinal case study: from Schon's model to self-directed learning. Journal of Continuing Education in the Health Professions. 2001;21(2):103.

Boreham NC. Modelling medical decision-making under uncertainty. British Journal of Educational Psychology. 1989;59(Pt 2):187.

Boreham NC, Mawer GE, Foster RW. Medical students' errors in pharmacotherapeutics. Medical education. 2000;34(3):188.

Borkhoff CM, Hawker GA, Kreder HJ, Glazier RH, Mahomed NN, Wright JG. Patients' gender affected physicians' clinical decisions when presented with standardized patients but not for matching paper patients. Journal of clinical epidemiology. 2009;62(5):527.

Borrell-Carrio F, Poveda BF, Seco EM, Castillejo JA, Gonzalez MP, Rodriguez EP. Family physicians' ability to detect a physical sign (hepatomegaly) from an unannounced standardized patient (incognito SP). European Journal of General Practice. 2011;17(2):95.

Bosk CL. Occupational rituals in patient management. New England Journal of Medicine. 1980;303(2):71.

Boulet JR, McKinley DW. Investigating gender-related construct-irrelevant components of scores on the written assessment exercise of a high-stakes certification assessment. Advances in Health Sciences Education. 2005;10(1):53.

Boulouffe C, Charlin B, Vanpee D. Evaluation of clinical reasoning in basic emergencies using a script concordance test. American Journal of Pharmaceutical Education. 2010;74(10):194.

Bouwmans AE, Weber WE. Neurologists' diagnostic accuracy of depression and cognitive problems in patients with parkinsonism. BMC Neurology. 2012;12:37.

Bowe CM, Voss J, Aretz HT. Case method teaching: an effective approach to integrate the basic and clinical sciences in the preclinical medical curriculum. Medical teacher. 2009;31(9):834.

Bowles KS. Effects of critical thinking, grade point average, and college experience on the clinical judgement skills of baccalaureate nursing students. 1997:104 p.

Braddock CH, Fihn SD, Levinson W, Jonsen AR, Pearlman RA. How doctors and patients discuss routine clinical decisions. Informed decision making in the outpatient setting. Journal of General Internal Medicine. 1997;12(6):339.

Brahler CJ, Quitadamo IJ, Johnson EC. Student critical thinking is enhanced by developing exercise prescriptions using online learning modules. Advances in Physiology Education. 2002;26(1-4):210.

Brannen ML, Cameron KA, Adler M, Goodman D, Holl JL. Admission handoff communications: clinician's shared understanding of patient severity of illness and problems. Journal of patient safety. 2009;5(4):237.

Brannick MT, Fabri PJ, Zayas-Castro J, Bryant RH. Evaluation of an error-reduction training program for surgical residents. Academic Medicine. 2009;84(12):1809.

Brien PGO. The relationship of female nurses' expertise to empathic concern, perspective taking, cognitive complexity, and analytic interactive style. 1992:142 p.

Briggs CL. What were they thinking? Nursing students' thought processes underlying pain management decisions. Nursing Education Perspectives. 2010;31(2):84.

Brillman JC, Doezema D, Tandberg D, Sklar DP, Skipper BJ. Does a physician visual assessment change triage? American Journal of Emergency Medicine. 1997;15(1):29.

Broderick GA, Abdolrasulnia M. Men's sexual health: evaluating the effectiveness of print- and PDA-based CME. Journal of Sexual Medicine. 2009;6(9):2417.

Brokaw JJ, Torbeck LJ, Bell MA, Deal DW. Impact of a competency-based curriculum on medical student advancement: a ten-year analysis. Teaching & Learning in Medicine. 2011;23(3):207.

Broyles IL, Cyr PR, Korsen N. Open book tests: assessment of academic learning in clerkships. Medical teacher. 2005;27(5):456.

Bruce CD, Robert C, Sid B, Mark F, Bruce S, Joseph SK, et al. Cognitive Elements in Clinical Decision-Making. Advances in Health Sciences Education. 2010;15(2):229.

Bruce S, Bridges EJ, Holcomb JB. Preparing to respond: Joint Trauma Training Center and USAF Nursing Warskills Simulation Laboratory. Critical Care Nursing Clinics of North America. 2003;15(2):149.

Bruin ABd, Schmidt HG, Rikers RM. The role of basic science knowledge and clinical knowledge in diagnostic reasoning: a structural equation modeling approach. Academic Medicine. 2005;80(8):765.

Brunt BA. Models, measurement, and strategies in developing critical-thinking skills. Journal of continuing education in nursing. 2005;36(6):255.

Brunt BA. Critical thinking in nursing: an integrated review. Journal of continuing education in nursing. 2005;36(2):60.

Bryson C. An examination of critical thinking in a self-care dialysis unit. Cannt Journal. 2003;13(1):34.

Buckingham CD. Psychological cue use and implications for a clinical decision support system. Medical Informatics & the Internet in Medicine. 2002;27(4):237.

Bucknall T, Manias E, Botti M. Acute pain management: implications of scientific evidence for nursing practice in the postoperative context. International journal of nursing practice. 2001;7(4):266.

Bucknall TK. Critical care nurses' decision-making activities in the natural clinical setting. Journal of Clinical Nursing. 2000;9(1):25.

Bull MJ. Using structured academic controversy with nursing students. Nurse educator. 2007;32(5):218.

Burke MD, Connelly DP. Systematic instruction in laboratory medicine. Effects on the clinical problem solving performance of medical students. Human pathology. 1981;12(2):134.

Buyse T, Lievens F. Situational judgment tests as a new tool for dental student selection. Journal of dental education. 2011;75(6):743.

Byrnes M, West S. Registered nurses' clinical reasoning abilities: a study of self perception. Australian Journal of Advanced Nursing. 2000;17(3):18.

Cahan A, Gilon D, Manor O, Paltiel O. Probabilistic reasoning and clinical decision-making: do doctors overestimate diagnostic probabilities? Qjm. 2003;96(10):763.

Callis AN, McCann AL, Schneiderman ED, Babler WJ, Lacy ES, Hale DS. Application of basic science to clinical problems: traditional vs. hybrid problem-based learning. Journal of dental education. 2010;74(10):1113.

Campbell ET. Clinical judgment and critical thinking abilities among senior nursing students: a comparative study. 1998:129 p.

Campbell G, Watters DA. Making decisions in emergency surgery. ANZ Journal of Surgery. 2013;83(6):429.

Campbell S, Carter B. Clinical judgement in children's nursing. Journal of Child Health Care. 1997;1(2):57.

Carlson J, Abel M, Bridges D, Tomkowiak J. The impact of a diagnostic reminder system on student clinical reasoning during simulated case studies. Simulation in Healthcare: The Journal of The Society for Medical Simulation. 2011;6(1):11.

Carter C, Gentry JA. Use of pop quizzes as an innovative strategy to promote critical thinking in nursing students. Nurse educator. 2000;25(4):155.

Casassus P, Hivon R, Gagnayre R, d'Ivernois JF. An initial experiment in haematology instruction using the problem-based learning method in third-year medical training in France. Hematology & Cell Therapy. 1999;41(4):137.

Case K, Harrison K, Roskell C. Differences in the clinical reasoning process of expert and novice cardiorespiratory physiotherapists. Physiotherapy. 2000;86(1):14.

Celia LM, Gordon PR. Using problem-based learning to promote critical thinking in an orientation program for novice nurses. Journal for Nurses in Staff Development - JNSD. 2001;17(1):12.

Chalabian J, Formenti S, Russell C, Pearce J, Dunnington G. Comprehensive needs assessment of clinical breast evaluation skills of primary care residents. Annals of Surgical Oncology. 1998;5(2):166.

Chamberland M, St-Onge C, Setrakian J, Lanthier L, Bergeron L, Bourget A, et al. The influence of medical students' self-explanations on diagnostic performance. Medical education. 2011;45(7):688.

Chambers DW. Preliminary evidence for a general competency hypothesis. Journal of dental education. 2001;65(11):1243.

Chambers DW. Lessons from students in a critical thinking course: a case for the third pedagogy. Journal of dental education. 2009;73(1):65.

Champlain AFD, Cuddy MM, Scoles PV, Brown M, Swanson DB, Holtzman K, et al. Progress testing in clinical science education: results of a pilot project between the National Board of Medical Examiners and a US Medical School. Medical teacher. 2010;32(6):503.

Chan CH, Donnelly MB, Caspary E. An approach to teaching and evaluating diagnostic reasoning. Research in medical education:proceedings of the annual Conference. 1986;25:129.

Charlin B, Lubarsky S, Millette B, Crevier F, Audetat MC, Charbonneau A, et al. Clinical reasoning processes: unravelling complexity through graphical representation. Medical education. 2012;46(5):454.

Chase SK. Clinical judgment by critical case nurses: an ethnographic study. 1990:199 p.

Chau JP, Chang AM, Lee IF, Ip WY, Lee DT, Wootton Y. Effects of using videotaped vignettes on enhancing students' critical thinking ability in a baccalaureate nursing programme. Journal of advanced nursing. 2001;36(1):112.

Chen H, Parker G, Kua J, Jorm A, Loh J. Mental health literacy in Singapore: a comparative survey of psychiatrists and primary health professionals. Annals of the Academy of Medicine, Singapore. 2000;29(4):467.

Chimowitz MI, Logigian EL, Caplan LR. The accuracy of bedside neurological diagnoses. Annals of Neurology. 1990;28(1):78.

Chipchase L, Prentice CE. Expert physiotherapists' clinical decision-making in acute care. Part one. International Journal of Therapy & Rehabilitation. 2006;13(8):343.

Choi BC, Jokovic A, Kay EJ, Main PA, Leake JL. Reducing variability in treatment decision-making: effectiveness of educating clinicians about uncertainty. Medical education. 1998;32(1):105.

Christie LA. Expertise in nurses' clinical judgments: the role of cognitive variables and experience. 1996:318 p.

Cianciolo AT, Williams RG, Klamen DL, Roberts NK. Biomedical knowledge, clinical cognition and diagnostic justification: a structural equation model. Medical education. 2013;47(3):309.

Cioffi JM, Swain J, Arundell F. The decision to suture after childbirth: cues, related factors, knowledge and experience used by midwives. Midwifery. 2010;26(2):246.

Cise JS, Wilson CS, Thie MJ. A qualitative tool for critical thinking skill development. Nurse educator. 2004;29(4):147.

Cisneros RM. Assessment of critical thinking in pharmacy students. American Journal of Pharmaceutical Education. 2009;73(4):66.

Clark RC. Clinical decision making by beginning nurses: a naturalistic study. 1996:103 p.

Clarke JR. Decision making in surgical practice. World journal of surgery. 1989;13(3):245.

Clayton LH. Concept mapping: an effective, active teaching-learning method. Nursing Education Perspectives. 2006;27(4):197.

Coderre S, Jenkins D, McLaughlin K. Qualitative differences in knowledge structure are associated with diagnostic performance in medical students. Advances in Health Sciences Education. 2009;14(5):677.

Coderre S, Mandin H, Harasym PH, Fick GH. Diagnostic reasoning strategies and diagnostic success. Medical education. 2003;37(8):695.

Coderre S, Wright B, McLaughlin K. To think is good: querying an initial hypothesis reduces diagnostic error in medical students. Academic Medicine. 2010;85(7):1125.

Collins AS, Graves BA, Gullette D, Edwards R. Developing an interactive microsimulation method in pharmacology. Journal of Nursing Education. 2010;49(7):410.

Collins LF, Affeldt J. Bridging the clinical reasoning gap. OT Practice. 1996;1(8):33.

Colliver JA, Swartz MH, Robbs RS. The effect of examinee and patient ethnicity in clinical-skills assessment with standardized patients. Advances in Health Sciences Education. 2001;6(1):5.

Colucciello ML. Critical thinking skills and dispositions of baccalaureate nursing students--a conceptual model for evaluation. Journal of Professional Nursing. 1997;13(4):236.

Comer SK. Patient care simulations: role playing to enhance clinical understanding. Nursing Education Perspectives. 2005;26(6):357.

Conn JJ, Lake FR, McColl GJ, Bilszta JL, Woodward-Kron R. Clinical teaching and learning: from theory and research to application. Medical Journal of Australia. 2012;196(8):527.

Connell KJ, Sinacore JM, Schmid FR, Chang RW, Perlman SG. Assessment of clinical competence of medical students by using standardized patients with musculoskeletal problems. Arthritis & Rheumatism. 1993;36(3):394.

Connors P. Assessing written evidence of critical thinking using an analytic rubric. Journal of Nutrition Education & Behavior. 2008;40(3):193.

Conradi E, Kavia S, Burden D, Rice A, Woodham L, Beaumont C, et al. Virtual patients in a virtual world: Training paramedic students for practice. Medical teacher. 2009;31(8):713.

Considine J, Ung L, Thomas S. Triage nurses' decisions using the National Triage Scale for Australian emergency departments. Accident & Emergency Nursing. 2000;8(4):201.

Considine J, Ung L, Thomas S. Clinical decisions using the National Triage Scale: how important is postgraduate education? Accident & Emergency Nursing. 2001;9(2):101.

Cook DA, Triola MM. Virtual patients: a critical literature review and proposed next steps. Medical education. 2009;43(4):303.

Cooper NJ. The use of narrative in the development of critical thinking. Nurse education today. 2000;20(7):513.

Cope MK, Baker HH, Foster RW, Boisvert CS. Relationships between clinical rotation subscores, COMLEX-USA examination results, and school-based performance measures. Journal of the American Osteopathic Association. 2007;107(11):502.

Corder JB. The association among critical thinking, clinical decision-making, and selected demographic characteristics of generic baccalaureate nursing students. 1992:128 p.

Cornelius FH. Handheld technology and nursing education: utilization of handheld technology in development of clinical decision-making in undergraduate nursing students. 2005:155 p.

Corrigan M, Reardon M, Shields C, Redmond H. "SURGENT" -- student e-learning for reality: the application of interactive visual images to problem-based learning in undergraduate surgery. Journal of Surgical Education. 2008;65(2):120.

Courteille O, Bergin R, Stockeld D, Ponzer S, Fors U. The use of a virtual patient case in an OSCE-based exam--a pilot study. Medical teacher. 2008;30(3):e66.

Cox KR. How did you guess? Or, what do multiple-choice questions measure? Medical Journal of Australia. 1976;1(23):884.

Cranley L, Doran DM. Nurses' integration of outcomes assessment data into practice. Outcomes Management. 2004;8(1):13.

Crebbin W, Beasley SW, Watters DA. Clinical decision making: how surgeons do it. ANZ Journal of Surgery. 2013;83(6):422.

Crespo KE, Torres JE, Recio ME. Reasoning process characteristics in the diagnostic skills of beginner, competent, and expert dentists. Journal of dental education. 2004;68(12):1235.

Critchley LA, Kumta SM, Ware J, Wong JW. Web-based formative assessment case studies: role in a final year medicine two-week anaesthesia course. Anaesthesia & Intensive Care. 2009;37(4):637.

Crites GE, Markert RJ, Goggans DS, Richardson WS. Local development of MCQ tests for evidence-based medicine and clinical decision making can be successful. Teaching & Learning in Medicine. 2012;24(4):341.

Croke E. The use of structured reflective journal questions to promote fundamental development of clinical decision-making abilities of the first-semester nursing student. Contemporary Nurse. 2004;17(1-2):125.

Croke EM. Role of reflective thinking in the development of clinical decision making in first-semester nursing students. 1999:343 p.

Croskerry P. Commentary: Lowly interns, more is merrier, and the Casablanca Strategy. Academic Medicine. 2011;86(1):8.

Croskerry P, Abbass A, Wu AW. Emotional influences in patient safety. Journal of patient safety. 2010;6(4):199.

Crossingham G, Gale T, Roberts M, Carr A, Langton J, Anderson I. Content validity of a clinical problem solving test for use in recruitment to the acute specialties. Clinical Medicine. 2011;11(1):23.

Crow R, Spicer J. Categorisation of the patient's medical condition--an analysis of nursing judgement. International journal of nursing studies. 1995;32(5):413.

Crowley RS, Naus GJ, Friedman CP. Development of visual diagnostic expertise in pathology. Proceedings / AMIA Annual Symposium. 2001:125.

Crowley RS, Naus GJ, Stewart J, Friedman CP. Development of visual diagnostic expertise in pathology -- an information-processing study. Journal of the American Medical Informatics Association. 2003;10(1):39.

Cruz FAD. Managing patient care: a substantive theory of clinical decision-making in home health care nursing. 1991:246 p.

Cullen DL. Clinical education and clinical evaluation of respiratory therapy students. Respiratory care clinics of North America. 2005;11(3):425.

Curran MJ, Campbell J, Rugg G. An investigation into the clinical reasoning of both expert and novice podiatrists. Foot. 2006;16(1):28.

Danerek M, Dykes A. Research. The meaning of problem solving in critical situations. British Journal of Midwifery. 2001;9(3):179.

Dare AJ, Cardinal A, Kolbe J, Bagg W. What can the history tell us? An argument for observed history-taking in the trainee intern long case assessment. New Zealand Medical Journal. 2008;121(1282):51.

DaRosa D, Rogers DA, Williams RG, Hauge LS, Sherman H, Murayama K, et al. Impact of a structured skills laboratory curriculum on surgery residents' intraoperative decision-making and technical skills. Academic Medicine. 2008;83(10 Suppl):S68.

DaRosa DA, Dawson-Saunders B, Folse R. A comparison of objective and subjective measures of clinical competence. Evaluation & Program Planning. 1985;8(4):327.

Davidson JE. Preceptor use of classroom assessment techniques to stimulate higher-order thinking in the clinical setting. Journal of continuing education in nursing. 2009;40(3):139.

Davies C, Howell D. A qualitative study: Clinical decision making in low back pain. Physiotherapy Theory and Practice. 2012;28(2):95.

Davis JK, Inamdar S, Stone RK. Assessment of knowledge and clinical judgment in the pediatric residency. Journal of medical education. 1985;60(6):468.

Davis LE, King MK. Assessment of medical student clinical competencies in the neurology clinic. Neurology. 2007;68(8):597.

Davis LE, King MK, Skipper BJ. Education research: Assessment of neurology resident clinical competencies in the neurology clinic. Neurology. 2009;72(1):e1.

Davis MH, Harden RM, Pitts NB. GDPs' and specialists' decisions in the management of fissure caries. Journal of dentistry. 1992;20(6):345.

Davis P, Russell AS, Skeith KJ. The use of standardized patients in the performance of a needs assessment and development of a CME intervention in rheumatology for primary care physicians. Journal of Rheumatology. 1997;24(10):1995.

Dawson-Saunders B, Mast TA, Finch WT, Konrad HR, Folse JR. Content knowledge and problem-solving skill in reviewing medical charts. Medical education. 1984;18(1):31.

Deborah AB, Nicholas JK, Celia FG, Myers JH. Evaluation of a diagnostic reasoning program (D × R): Exploring student perceptions and addressing faculty concerns. Journal of Interactive Media in Education. 1998;98(1).

Deborah D. Measuring the Problem-Solving Ability of Students and Residents by Microcomputer. Journal of medical education. 1986;61(6):461.

Dejano TS. An appraisal of medical students' reflection-in-learning. Medical education. 2000;34(3):182.

Demner-Fushman D, Chapman WW, McDonald CJ. What can natural language processing do for clinical decision support? Journal of Biomedical Informatics. 2009;42(5):760.

Dequeker J, Jaspaert R. Teaching problem-solving and clinical reasoning: 20 years experience with video-supported small-group learning. Medical education. 1998;32(4):384.

Deschenes MF, Charlin B, Gagnon R, Goudreau J. Use of a script concordance test to assess development of clinical reasoning in nursing students. Journal of Nursing Education. 2011;50(7):381.

DeSimone BB. Curriculum design to promote the critical thinking of accelerated bachelor's degree nursing students. Nurse educator. 2006;31(5):213.

Devitt P, Palmer E. Computers in medical education 1: evaluation of a problem-orientated learning package. Australian & New Zealand Journal of Surgery. 1998;68(4):284.

Dexter P, Applegate M, Backer J, Claytor K, Keffer J, Norton B, et al. A proposed framework for teaching and evaluating critical thinking in nursing. Journal of Professional Nursing. 1997;13(3):160.

Dickerson PS. Nurturing critical thinkers. Journal of continuing education in nursing. 2005;36(2):68.

Dickieson P, Carter LM, Walsh M. Integrative thinking and learning in undergraduate nursing education: three strategies. International Journal of Nursing Education Scholarship. 2008;5:Arte.

Dielman ET. Psychometric Properties of Clinical Performance Ratings. Evaluation and the Health Professions. 1980;3(1):103.

Dillard N, Sideras S, Ryan M, Carlton KH, Lasater K, Siktberg L. A collaborative project to apply and evaluate the clinical judgment model through simulation. Nursing Education Perspectives. 2009;30(2):99.

Dincher JR, Stidger SL. Evaluation of a written simulation format for clinical nursing judgment: a pilot study. Nursing research. 1976;25(4):280.

Diserens D, Schwartz MW, Guenin M, Taylor LA. Measuring the problem-solving ability of students and residents by microcomputer. Journal of medical education. 1986;61(6):461.

Dobrzykowski TM. Teaching strategies to promote critical thinking skills in nursing staff. Journal of continuing education in nursing. 1994;25(6):272.

Doig K. Focus: current issues in assessment. Common formats of multiple choice questions testing analytical skills in clinical laboratory science. Clinical Laboratory Science. 2000;13(1):40.

Don ED. Heuristics and Biases in Medical Decision-Making. Journal of Medical Education. 1978;53(8):682.

Donald EM, Gerard FD, David BS. Medical Licensing Examinations in the United States. Journal of dental education. 2002;66(5):595.

Donnelly MB, Sisson JC, Woolliscroft JO. The reliability of a hypothesis generation and testing task. Medical education. 1990;24(6):507.

Donnon T, Violato C. Medical students' clinical reasoning skills as a function of basic science achievement and clinical competency measures: a structural equation model. Academic Medicine. 2006;81(10 Suppl):S120.

Doody C, McAteer M. Clinical reasoning of expert and novice physiotherapists in an outpatient orthopaedic setting. Physiotherapy. 2002;88(5):258.

Doran T, Maudsley G, Zakhour H. Time to think? Questionnaire survey of pre-registration house officers' experiences of critical appraisal in the Mersey Deanery. Medical education. 2007;41(5):487.

Dore KL, Brooks LR, Weaver B, Norman GR. Influence of familiar features on diagnosis: instantiated features in an applied setting. Journal of Experimental Psychology: Applied. 2012;18(1):109.

Dory V, Gagnon R, Charlin B. Is case-specificity content-specificity? An analysis of data from extended-matching questions. Advances in Health Sciences Education. 2010;15(1):55.

Dory V, Gagnon R, Vanpee D, Charlin B. How to construct and implement script concordance tests: insights from a systematic review. Medical education. 2012;46(6):552.

Dowding D. Examining the effects that manipulating information given in the change of shift report has on nurses' care planning ability. Journal of advanced nursing. 2001;33(6):836.

Dowling S, Bliss LS. Cognitive complexity, rhetorical sensitivity: contributing factors in clinical skill? Journal of communication disorders. 1984;17(1):9.

Downar J, Bhatt M, Montague PR. Neural correlates of effective learning in experienced medical decision-makers. PLoS ONE [Electronic Resource]. 2011;6(11):e27768.

Drennan J. Critical thinking as an outcome of a Master's degree in Nursing programme. Journal of advanced nursing. 2010;66(2):422.

Driver JM, Nelson TG, Simpson R, Wall C. To refer or not to refer: a qualitative study of reasons for referral from Role 1. Journal of the Royal Army Medical Corps. 2012;158(3):208.

Duff B. A theoretically informed education program designed specifically for acute surgical nurses. Nurse education today. 2012;32(8):e73.

Dunkley-Bent J, Jones D. An evaluation of clinical decision making study days. British Journal of Midwifery. 2010;18(5):302.

Dunn MM, Woolliscroft JO. Assessment of a surgical pattern recognition examination. American Journal of Surgery. 1995;169(3):341.

Dunphy BC, Cantwell R, Bourke S, Fleming M, Smith B, Joseph KS, et al. Cognitive elements in clinical decision-making: toward a cognitive model for medical education and understanding clinical reasoning. Advances in Health Sciences Education. 2010;15(2):229.

Durak HI, Caliskan SA, Bor S, Vleuten CVd. Use of case-based exams as an instructional teaching tool to teach clinical reasoning. Medical teacher. 2007;29(6):e170.

Durning S, Artino AR, Pangaro L, Vleuten CPvd, Schuwirth L. Context and clinical reasoning: understanding the perspective of the expert's voice. Medical education. 2011;45(9):927.

Durning SJ, Artino A, Boulet J, Rochelle JL, Vleuten CVd, Arze B, et al. The feasibility, reliability, and validity of a post-encounter form for evaluating clinical reasoning. Medical teacher. 2012;34(1):30.

Durning SJ, Artino AR, Beckman TJ, Graner J, Vleuten Cvd, Holmboe E, et al. Does the think-aloud protocol reflect thinking? Exploring functional neuroimaging differences with thinking (answering multiple choice questions) versus thinking aloud. Medical teacher. 2013;35(9):720.

Durning SJ, Artino AR, Boulet JR, Dorrance K, Vleuten Cvd, Schuwirth L. The impact of selected contextual factors on experts' clinical reasoning performance (does context impact clinical reasoning performance in experts?). Advances in Health Sciences Education. 2012;17(1):65.

Durning SJ, Artino AR, Schuwirth L, Vleuten Cvd. Clarifying assumptions to enhance our understanding and assessment of clinical reasoning. Academic Medicine. 2013;88(4):442.

Durning SJ, Dong T, Artino AR, LaRochelle J, Pangaro LN, Vleuten Cvd, et al. Instructional authenticity and clinical reasoning in undergraduate medical education: a 2-year, prospective, randomized trial. Military medicine. 2012;177(9 Suppl):38.

Durning SJ, Graner J, Artino AR, Pangaro LN, Beckman T, Holmboe E, et al. Using functional neuroimaging combined with a think-aloud protocol to explore clinical reasoning expertise in internal medicine. Military medicine. 2012;177(9 Suppl):72.

Dyches CS. Clinical teaching and clinical reasoning in a baccalaureate degree nursing program. 1998:83 p.

Eagle CJ, Martineau R, Hamilton K. The oral examination in anaesthetic resident evaluation. Canadian Journal of Anaesthesia. 1993;40(10):947.

Eberhard J, Klomp HJ, Foge M, Hedderich J, Schmidt HG. The intermediate effect and the diagnostic accuracy in clinical case recall of students and experts in dental medicine. European Journal of Dental Education. 2009;13(3):128.

Ebert MH, Faulkner L, Stubbe DE, Winstead DK. Maintenance of certification in psychiatry. Journal of Clinical Psychiatry. 2009;70(10):e39.

Eckerblad J, Eriksson H, Karner A, Edell-Gustafsson U. Nurses' conceptions of facilitative strategies of weaning patients from mechanical ventilation--a phenomenographic study. Intensive & Critical Care Nursing. 2009;25(5):225.

Edelen BG. Measuring and enhancing clinical decision-making ability among students in an Associate Degree Nursing program. 2009:174 p.

Eerden KV. Using critical thinking vignettes to evaluate student learning. Nursing & Health Care Perspectives. 2001;22(5):231.

Egerod I. Uncertain terms of sedation in ICU. How nurses and physicians manage and describe sedation for mechanically ventilated patients. Journal of Clinical Nursing. 2002;11(6):831.

Ekwo EE, Loening-Baucke V. Clinical problem solving and clinical knowledge. Medical education. 1979;13(4):251.

Elenita F, Kristina Z, Hå H, Uno F. Clinical reasoning in nursing, a think-aloud study using virtual patients – A base for an innovative assessment. Nurse education today. 2014;34(4):538.

Eli I. Reducing confirmation bias in clinical decision-making. Journal of dental education. 1996;60(10):831.

Ellen C, Cathy E, Steven T. Clinical Decision Making in the Acute Care Environment: A Survey of Practicing Clinicians. Journal of Acute Care Physical Therapy (Acute Care Section - APTA, Inc). 2011;2(2):46.

Ellen O. Situated Cognition: Its Relationship to Simulation in Nursing Education. Clinical Simulation in Nursing. 2012;8(7):e273.

Ellis PA. Clinical decision-making: a process. 1993:155 p.

Elstein AS. Beyond multiple-choice questions and essays: the need for a new way to assess clinical competence. Academic Medicine. 1993;68(4):244.

Endicott J. Good diagnoses require good diagnosticians: collecting and integrating the data. American Journal of Medical Genetics. 2001;105(1):48.

Ericsson KA. An expert-performance perspective of research on medical expertise: the study of clinical performance. Medical education. 2007;41(12):1124.

Eta SB. A New Approach to Evaluating Problem-Solving in Medical Students. Journal of Medical Education. 1974;49(7):666.

Eugène JFMC, Henny PAB, Henk GS. The role of illness scripts in the development of medical diagnostic expertise: Results from an interview study. Cognition and Instruction. 1998;16(4):367.

Evered A, Walker D, Watt AA, Perham N. To what extent does nonanalytic reasoning contribute to visual learning in cytopathology? Cancer Cytopathology. 2013;121(6):329.

Faucher C, Tardif J, Chamberland M. Optometrists' clinical reasoning made explicit: a qualitative study. Optometry & Vision Science. 2012;89(12):1774.

Ferguson LM. A grounded theory study of the new nurse's journey toward competence in clinical judgement. 2006:358 p.

Ferrario CG. The association of clinical experience and emergency nurses' diagnostic reasoning. 2001:227 p.

Fesler-Birch DM. Critical thinking and patient outcomes: a review. Nursing outlook. 2005;53(2):59.

Fine JMB. A descriptive analysis of the clinical reasoning of expert labor and delivery nurses. 1997:342 p.

Finlay K, Norman GR, Keane DR, Stolberg H. A web-based test of residents' skills in diagnostic radiology. Canadian Association of Radiologists Journal. 2006;57(2):106.

Fischer MR, Kopp V, Holzer M, Ruderich F, Junger J. A modified electronic key feature examination for undergraduate medical students: validation threats and opportunities. Medical teacher. 2005;27(5):450.

Fletcher G, Flin R, McGeorge P, Glavin R, Maran N, Patey R. Anaesthetists' Non-Technical Skills (ANTS): evaluation of a behavioural marker system. British journal of anaesthesia. 2003;90(5):580.

Follman J. Research on nurses. Nurse educator. 2003;28(6):255.

Fonteyn ME. A descriptive analysis of expert critical care nurses' clinical reasoning. 1991:280 p.

Fowler LP. Clinical reasoning of home health nurses: a verbal protocol analysis. 1994:147 p.

Fox MC, Ericsson KA, Best R. Do Procedures for Verbal Reporting of Thinking Have to Be Reactive? A Meta-Analysis and Recommendations for Best Reporting Methods. Psychological bulletin. 2011;137(2):316.

Frank D, Ronald G, Jonathan C. Changing test ordering behavior: A randomized controlled trial comparing probabilistic reasoning with cost-containment education. Medical care. 1989;27(1):45.

Friedman CP, France CL, Drossman DD. A randomized comparison of alternative formats for clinical simulations. Medical Decision Making. 1991;11(4):265.

Friedman CP, Gatti GG, Franz TM, Murphy GC, Wolf FM, Heckerling PS, et al. Do physicians know when their diagnoses are correct? Implications for decision support and error reduction. Journal of General Internal Medicine. 2005;20(4):334.

Fromme HB, Karani R, Downing SM. Direct observation in medical education: a review of the literature and evidence for validity. Mount Sinai Journal of Medicine. 2009;76(4):365.

Frye B, Alfred N, Campbell M. Use of the Watson-Glaser Critical Thinking Appraisal with BSN students. Nursing & Health Care Perspectives. 1999;20(5):253.

Gay S, Bartlett M, McKinley R. Teaching clinical reasoning to medical students. The clinical teacher. 2013;10(5):308.

Geigle PR. What do physical therapy educators define as clinical decision-making and how are these definitions evidenced in their teaching? 2002:107 p.

Geoffrey RN. Reliability and construct validity of some cognitive measures of clinical reasoning. Teaching and learning in medicine. 1989;1(4):194.

Gillespie M. Using the Situated Clinical Decision-Making framework to guide analysis of nurses' clinical decision-making. Nurse Education in Practice. 2010;10(6):333.

Girot EA. Assessment of competence in clinical practice: a phenomenological approach. Journal of advanced nursing. 1993;18(1):114.

Glavin-Spiehs C. The differences between field dependence/independence and two measurements of clinical judgment ability in senior baccalaureate nursing students. 1991:207 p.

Glenn AM. Consultation Letters as a Method for Assessing In-Training Performance in a Department of Medicine. Evaluation and the Health Professions. 1988;11(1):21.

Gonzalez MP, Fernandez EM, Castillejo JAP, Jimenez FQ, Pitz PB, Arrabal CI. [Evaluation of clinical competence of family medicine trainer]. Atencion Primaria. 2004;34(2):68.

Goulet F, Jacques A, Gagnon R, Charlin B, Shabah A. Poorly performing physicians: does the Script Concordance Test detect bad clinical reasoning? Journal of Continuing Education in the Health Professions. 2010;30(3):161.

Graaff Ed. A test of medical problem-solving scored by nurses and doctors: the handicap of expertise. Medical education. 1989;23(4):381.

Graaff Ed, Post GJ, Drop MJ. Validation of a new measure of clinical problem-solving. Medical education. 1987;21(3):213.

Graber ML, Kissam S, Payne VL, Meyer AN, Sorensen A, Lenfestey N, et al. Cognitive interventions to reduce diagnostic error: a narrative review. BMJ Quality & Safety. 2012;21(7):535.

Groenier M, Pieters JM, Hulshof CD, Wilhelm P, Witteman CLM. Psychologists' judgements of diagnostic activities: Deviations from a theoretical model. Clinical Psychology and Psychotherapy. 2008;15(4):256.

Groves M, Dick ML, McColl G, Bilszta J. Analysing clinical reasoning characteristics using a combined methods approach. BMC Medical Education. 2013;13:144.

Gruppen LD, Palchik NS, Wolf FM, Laing TJ, Oh MS, Davis WK. Medical student use of history and physical information in diagnostic reasoning. Arthritis Care & Research. 1993;6(2):64.

Gruppen LD, Woolliscroft JO, Wolf FM. The contribution of different components of the clinical encounter in generating and eliminating diagnostic hypotheses. Research in medical education:proceedings of the annual Conference. 1988;27:242.

Haffer AG. Beginning nurses' diagnostic reasoning behaviors derived from observation and verbal protocol analysis. 1990:252 p.

Hageman MG, Guitton TG, Ring D, Group Science of V. How surgeons make decisions when the evidence is inconclusive. Journal of Hand Surgery - American Volume. 2013;38(6):1202.

Haley S. Comparing Lasater's Clinical Judgment Rubric scores across faculty, self-assessment, & outcome scores. 2013:88 p.

Harasym P, Baumber J, Bryant H, Fundytus D, Preshaw R, Watanabe M, et al. An evaluation of the clinical problem-solving process using a simulation technique. Medical education. 1980;14(6):381.

Hardin LE. Cognitive processes of second-year veterinary students in clinical case resolution. Journal of Veterinary Medical Education. 2003;30(3):236.

Harjai PK, Tiwari R. Model of critical diagnostic reasoning: achieving expert clinician performance. Nursing Education Perspectives. 2009;30(5):305.

Harper AC, Roy WB, Norman GR, Rand CA, Feightner JW. Difficulties in clinical skills evaluation. Medical education. 1983;17(1):24.

Hartley RC. Development of a cognitive self-report using the ACT* theory of cognitive skill acquisition. 1992:204 p.

Hassebrock F, Johnson PE, Bullemer P, Fox PW, Moller JH. When less is more: representation and selective memory in expert problem solving. American Journal of Psychology. 1993;106(2):155.

Hatala R, Cole G, Kassen BO, Bacchus CM, Issenberg SB. Does physical examination competence correlate with bedside diagnostic acumen? An observational study. Medical teacher. 2007;29(2-3):199.

Hatala R, Norman GR, Brooks LR. Impact of a clinical scenario on accuracy of electrocardiogram interpretation. Journal of General Internal Medicine. 1999;14(2):126.

Heemskerk L, Norman G, Chou S, Mintz M, Mandin H, McLaughlin K. The effect of question format and task difficulty on reasoning strategies and diagnostic performance in Internal Medicine residents. Advances in Health Sciences Education. 2008;13(4):453.

Hennen BK. Measuring the complexity of clinical problems. Journal of medical education. 1984;59(6):487.

Henry SB. The effect of level of patient acuity on clinical decision-making by critical care nurses with varying levels of knowledge and experience. 1989:200 p.

Higuchi KAS. Professional nursing education: Cognitive processes utilized in clinical decision making. 1997:156 p.

Hobus PP, Schmidt HG, Boshuizen HP, Patel VL. Contextual factors in the activation of first diagnostic hypotheses: expert-novice differences. Medical education. 1987;21(6):471.

Hodges B, Regehr G, McNaughton N, Tiberius R, Hanson M. OSCE checklists do not capture increasing levels of expertise. Academic Medicine. 1999;74(10):1129.

Hoffman KA, Aitken LM, Duffield C. A comparison of novice and expert nurses' cue collection during clinical decision-making: verbal protocol analysis. International journal of nursing studies. 2009;46(10):1335.

Holmboe ES, Hawkins RE. Methods for evaluating the clinical competence of residents in internal medicine: a review. Annals of Internal Medicine. 1998;129(1):42.

Holzemer WL. The structure of problem solving in simulations. Nursing research. 1986;35(4):231.

Huffstutler SAY. Clinical decision-making processes of novice female baccalaureate and associate degree nurses. 1993:147 p.

Huhn K, Black L, Jensen GM, Deutsch JE. Construct validity of the Health Science Reasoning Test. Journal of allied health. 2011;40(4):181.

Humbert AJ, Besinger B, Miech EJ. Assessing clinical reasoning skills in scenarios of uncertainty: convergent validity for a Script Concordance Test in an emergency medicine clerkship and residency. Academic Emergency Medicine. 2011;18(6):627.

Humbert AJ, Johnson MT, Miech E, Friedberg F, Grackin JA, Seidman PA. Assessment of clinical reasoning: A Script Concordance test designed for pre-clinical medical students. Medical teacher. 2011;33(6):472.

Hurtz GM, Chinn RN, Barnhill GC, Hertz NR. Measuring clinical decision making: do key features problems measure higher level cognitive processes? Evaluation & the health professions. 2012;35(4):396.

Ilgen JS, Bowen JL, McIntyre LA, Banh KV, Barnes D, Coates WC, et al. Comparing diagnostic performance and the utility of clinical vignette-based assessment under testing conditions designed to encourage either automatic or analytic thought. Academic Medicine. 2013;88(10):1545.

Ilgen JS, Humbert AJ, Kuhn G, Hansen ML, Norman GR, Eva KW, et al. Assessing diagnostic reasoning: a consensus statement summarizing theory, practice, and future needs. Academic Emergency Medicine. 2012;19(12):1454.

Ingalls JR. ADN students' and their clinical nursing educators' correlates of perceived clinical decision-making. 1996:216 p.

Itano JK. A comparison of the clinical judgment process in experienced registered nurses and student nurses. Journal of Nursing Education. 1989;28(3):120.

Jane F. Time is the Essence: A Grounded Theory Study of Registered Nurse's Experiences with Clinical Reasoning. 2013:175 p.

Janet G. Some Cognitive Components of the Diagnostic Thinking Process. British Journal of Educational Psychology. 1982;52.

Janing J. Assessment of a scenario-based approach to facilitating critical thinking among paramedic students. Prehospital & Disaster Medicine. 1997;12(3):215.

Jensen GM, Givens D. Clinical reasoning: linking theory to practice and practice to theory. Neurology Report. 1999;23(4):137.

Jensen R. Clinical reasoning during simulation: comparison of student and faculty ratings. Nurse Education in Practice. 2013;13(1):23.

Jeremy LW, Robert MN, Lawrence MJT. Uses and misuses of thresholds in diagnostic decision making. Academic Medicine. 2010;85(3):556.

Jessica C. Why nurses should use clinical reasoning to diagnose a cough. Primary Health Care. 2013;23(7):18.

Joanna A. Exploring dental hygiene clinical decision making- a mixed methods study of potential organizational explanations: Phase I. Canadian Journal of Dental Hygiene. 2012;46(4):207.

Johannsson SL, Wertenberger DH. Using simulation to test critical thinking skills of nursing students. Nurse education today. 1996;16(5):323.

John ED, Heidi C, Russell W, Swapan C, Anju R. Information-Gathering Patterns Associated with Higher Rates of Diagnostic Error. Advances in Health Sciences Education. 2009;14(5):697.

Johnsen DC, Finkelstein MW, Marshall TA, Chalkley YM. A model for critical thinking measurement of dental student performance. Journal of dental education. 2009;73(2):177.

Johnsen DC, Lipp MJ, Finkelstein MW, Cunningham-Ford MA. Guiding dental student learning and assessing performance in critical thinking with analysis of emerging strategies. Journal of dental education. 2012;76(12):1548.

Johnson EA, Lasater K, Hodson-Carlton K, Siktberg L, Sideras S, Dillard N. Geriatrics in simulation: role modeling and clinical judgment effect. Nursing Education Perspectives. 2012;33(3):176.

Johnson G, Flagler S. Web-based unfolding cases: a strategy to enhance and evaluate clinical reasoning skills. Journal of Nursing Education. 2013;52(10):589.

Johnson LA. Quantifying the measurement of differential diagnosis. Medinfo. 1995;8(Pt 2):1294.

Jong Zd, Nies JAv, Peters SW, Vink S, Dekker FW, Scherpbier A. Interactive seminars or small group tutorials in preclinical medical education: results of a randomized controlled trial. BMC Medical Education. 2010;10:79.

Joorabchi B. Medical information processing skills: guide posts to clinical assessment. Medical teacher. 1989;11(3-4):331.

Jorge E, Marianne B, Oliver T. Script concordance test: Insights from the literature and early stages of its implementation in osteopathy. International Journal of Osteopathic Medicine. 2013;16(4):231.

Judith GC. Evaluating Medical Students' Patient Interviewing Skills: A Biopsychosocial Model. Evaluation and program planning. 1987;10(1):3.

Juul DH, Noe MJ, Nerenberg RL. A factor analytic study of branching patient management problems. Medical education. 1979;13(3):199.

Kaddoura MA. New graduate nurses' perceptions of the effects of clinical simulation on their critical thinking, learning, and confidence. Journal of continuing education in nursing. 2010;41(11):506.

Kamilah B. An exploration of the relationship between clinical decision-making ability and educational preparation among new graduate nurses. 2013:80 p.

Kanter SL, Brosenitsch TA, Mahoney JF, Staszewski J. Defining the correctness of a diagnosis: differential judgments and expert knowledge. Advances in Health Sciences Education. 2010;15(1):65.

Karen G. An investigation into the relationship between critical thinking skills and clinical judgment in the nurse practitioner student. 2010:181 p.

Karen G, Janice H. Challenges of Assessing Critical Thinking and Clinical Judgment in Nurse Practitioner Students. Journal of Nursing Education. 2014;53(3):S26.

Kassirer JP. Teaching clinical reasoning: case-based and coached. Academic Medicine. 2010;85(7):1118.

Kautz D, Kuiper R, Bartlett R, Buck R, Williams R, Knight-Brown P. Building evidence for the development of clinical reasoning using a rating tool with the Outcome-Present State-Test (OPT) Model. Southern Online Journal of Nursing Research. 2009;9(1):8p.

Kautz DD, Kuiper R, Pesut DJ, Knight-Brown P, Daneker D. Promoting clinical reasoning in undergraduate nursing students: application and evaluation of the Outcome Present State Test (OPT) model of clinical reasoning. International Journal of Nursing Education Scholarship. 2005;2:Arte.

Kawashima A, Petrini MA. Study of critical thinking skills in nursing students and nurses in Japan. Nurse education today. 2004;24(4):286.

Ken C. Perceiving clinical evidence. Medical education. 2002;36(12):1189.

Kennison MM. The evaluation of students' reflective writing for evidence of critical thinking. Nursing Education Perspectives. 2006;27(5):269.

Kent JD. Assessment of Knowledge and Clinical Judgment in the Pediatric Residency. Journal of medical education. 1985;60(6):468.

Kevin M, Remy MR, Henk GS. Is Analytic Information Processing a Feature of Expertise in Medicine? Advances in Health Sciences Education. 2008;13(1):123.

Khatami S, MacEntee MI, Pratt DD, Collins JB. Clinical reasoning in dentistry: a conceptual framework for dental education. Journal of dental education. 2012;76(9):1116.

Kiesewetter J, Ebersbach R, Gorlitz A, Holzer M, Fischer MR, Schmidmaier R. Cognitive problem solving patterns of medical students correlate with success in diagnostic case solutions. PLoS ONE [Electronic Resource]. 2013;8(8):e71486.

King CA, Bithell C. Expertise in diagnostic reasoning: a comparative study. British Journal of Therapy & Rehabilitation. 1998;5(2):78.

King G, Currie M, Bartlett DJ, Gilpin M, Willoughby C, Tucker MA, et al. The development of expertise in pediatric rehabilitation therapists: changes in approach, self-knowledge, and use of enabling and customizing strategies. Developmental neurorehabilitation. 2007;10(3):223.

King PL. A consideration of diagnostic reasoning skills in nurse practitioners-measures and influences. 2006:159 p.

Kinnaman ML. Exploring the clinical decision-making strategies of nurses. 2006:232 p.

Kogan JR, Conforti L, Bernabeo E, Iobst W, Holmboe E. Opening the black box of clinical skills assessment via observation: a conceptual model. Medical education. 2011;45(10):1048.

Kogan JR, Holmboe ES, Hauer KE. Tools for direct observation and assessment of clinical skills of medical trainees: a systematic review. JAMA. 2009;302(12):1316.

Kovacs G, Croskerry P. Clinical decision making: an emergency medicine perspective. Academic Emergency Medicine. 1999;6(9):947.

Kreiter CD, Bergus G. The validity of performance-based measures of clinical reasoning and alternative approaches. Medical education. 2009;43(4):320.

Krupat E, Sprague JM, Wolpaw D, Haidet P, Hatem D, Brien BO. Thinking critically about critical thinking: ability, disposition or both? Medical education. 2011;45(6):625.

Krupat E, Sprague JM, Wolpaw D, Haidet P, Hatem D, Brien BO. Thinking critically about critical thinking: Ability, disposition or both? Medical education. 2011;45(6):625.

Kuiper R, Heinrich C, Matthias A, Graham MJ, Bell-Kotwall L. Debriefing with the OPT model of clinical reasoning during high fidelity patient simulation. International Journal of Nursing Education Scholarship. 2008;5:Arte17.

Kuiper RA. Integration of innovative clinical reasoning pedagogies into a baccalaureate nursing curriculum. Creative nursing. 2013;19(3):128.

Kulatunga-Moruzi C, Brooks LR, Norman GR. Teaching posttraining: influencing diagnostic strategy with instructions at test. Journal of Experimental Psychology: Applied. 2011;17(3):195.

Labasky SM. A study of the relationships among student nurse epistemology, critical thinking, and clinical decision-making. 2004:147 p.

Ladyshewsky RK. Impact of peer-coaching on the clinical reasoning of the novice practitioner... including commentary by Richardson J. Physiotherapy Canada. 2004;56(1):15.

Laidig JM. Problem finding, problem defining, and problem solving by novice clinical teachers in nursing. 1995:367 p.

LaRochelle JS, Durning SJ, Pangaro LN, Artino AR, Vleuten Cvd, Schuwirth L. Impact of increased authenticity in instructional format on preclerkship students' performance: a two-year, prospective, randomized study. Academic Medicine. 2012;87(10):1341.

Larsson W, Lundberg N, Hillergard K. Use your good judgement - Radiographers' knowledge in image production work. Radiography. 2009;15(3):e11; e21.

Lasater K. The impact of high fidelity simulation on the development of clinical judgment in nursing students: an exploratory study. 2005:227 p.

Lasater K. Clinical judgment: the last frontier for evaluation. Nurse Education in Practice. 2011;11(2):86.

Lasater K, Nielsen A. Reflective journaling for clinical judgment development and evaluation. Journal of Nursing Education. 2009;48(1):40.

Lauri S, Salantera S. Developing an instrument to measure and describe clinical decision making in different nursing fields. Journal of Professional Nursing. 2002;18(2):93.

LeBlanc VR, MacDonald RD, McArthur B, King K, Lepine T. Paramedic performance in calculating drug dosages following stressful scenarios in a human patient simulator. Prehospital Emergency Care. 2005;9(4):439.

Lee AG, Oetting T, Beaver HA, Carter K, Ophthalmology Task Force on the ACatUoIDo. The ACGME Outcome Project in ophthalmology: practical recommendations for overcoming the barriers to local implementation of the national mandate. Survey of ophthalmology. 2009;54(4):507.

Lee JY, Mucksavage P, Kerbl DC, Osann KE, Winfield HN, Kahol K, et al. Laparoscopic warm-up exercises improve performance of senior-level trainees during laparoscopic renal surgery. Journal of Endourology. 2012;26(5):545.

Lettus MK, Moessner PH, Dooley L. The clinical portfolio as an assessment tool. Nursing administration quarterly. 2001;25(2):74.

Lewkonia RM, Harasym PH, Darwish HZ. Early introduction to medical problem-solving. Medical teacher. 1993;15(1):57.

Liaw JJ. Use of a training program to enhance NICU nurses' cognitive abilities for assessing preterm infant behaviors and offering supportive interventions. Journal of Nursing Research. 2003;11(2):82.

Lim EC, Seet RC, Oh VM, Chia BL, Aw M, Quak SH, et al. Computer-based testing of the modified essay question: the Singapore experience. Medical teacher. 2007;29(9):e261.

Lina K. Clinical judgment among new nursing graduates: A multiple-case study. 2010:330 p.

Linda R. Clinical Reasoning, Part 1: the Nature of Problem Solving, a Literature Review. British Journal of Occupational Therapy. 1996;59(4):178.

Linda R. Clinical Reasoning, Part 2: Novice/Expert Differences. British Journal of Occupational Therapy. 1996;59(5):212.

Lipner RS, Messenger JC, Kangilaski R, Baim DS, Holmes DR, Williams DO, et al. A technical and cognitive skills evaluation of performance in interventional cardiology procedures using medical simulation. Simulation in Healthcare: The Journal of The Society for Medical Simulation. 2010;5(2):65.

Long DM. Student understanding and use of clinical reasoning skills in occupational therapy: a phenomenological study. 2008:718 p.

Long MC, Redding BA. Evaluating clinical skills of RN students. Nurse educator. 1991;16(3):31.

Loomis J. Evaluating clinical competence of physical therapy students. Part 1: the development of an instrument. Physiotherapy Canada. 1985;37(2):83.

Lovegrove J, Hatfield D. The use of skills inventories to assess and grade practice: Part 1--design and implementation. Nurse Education in Practice. 2012;12(3):127.

Lubarsky S, Chalk C, Kazitani D, Gagnon R, Charlin B. The Script Concordance Test: a new tool assessing clinical judgement in neurology. Canadian Journal of Neurological Sciences. 2009;36(3):326.

Lubarsky S, Charlin B, Cook DA, Chalk C, Vleuten CPvd. Script concordance testing: a review of published validity evidence. Medical education. 2011;45(4):329.

Lubarsky S, Dory V, Duggan P, Gagnon R, Charlin B. Script concordance testing: from theory to practice: AMEE guide no. 75. Medical teacher. 2013;35(3):184.

MacKinnon GE, Pitterle ME, Boh LE, DeMuth JE. Computer-based patient simulations: hospital pharmacists' performance and opinions. American Journal of Hospital Pharmacy. 1992;49(11):2740.

Maebius NK. Relationships among critical thinking ability, locus-of-control, and clinical problem-solving performance of registered nurses. 1990:185 p.

Major DA. OSCEs--seven years on the bandwagon: the progress of an objective structured clinical evaluation programme. Nurse education today. 2005;25(6):442.

Mamede S, Gog Tv, Berge Kvd, Rikers RM, Saase JLv, Guldener Cv, et al. Effect of availability bias and reflective reasoning on diagnostic accuracy among internal medicine residents. JAMA. 2010;304(11):1198.

Mamede S, Gog Tv, Berge Kvd, Saase JLv, Schmidt HG. Why do doctors make mistakes? A study of the role of salient distracting clinical features. Academic Medicine. 2014;89(1):114.

Mamede S, Gog Tv, Sampaio AM, Faria RMd, Maria JP, Schmidt HG. How can students' diagnostic competence benefit most from practice with clinical cases? The effects of structured reflection on future diagnosis of the same and novel diseases. Academic Medicine. 2014;89(1):121.

Mamede S, Schmidt HG, Rikers RM, Penaforte JC, Coelho-Filho JM. Breaking down automaticity: case ambiguity and the shift to reflective approaches in clinical reasoning. Medical education. 2007;41(12):1185.

Mamede S, Splinter TA, Gog Tv, Rikers RM, Schmidt HG. Exploring the role of salient distracting clinical features in the emergence of diagnostic errors and the mechanisms through which reflection counteracts mistakes. BMJ Quality & Safety. 2012;21(4):295.

Marcia AB. Accreditation of Predoctoral Dental Education: Clinical Outcomes Assessment. Journal of dental education. 1991;55(11):729.

Marcum JA. An integrated model of clinical reasoning: dual-process theory of cognition and metacognition. Journal of evaluation in clinical practice. 2012;18(5):954.

Mark VP, Stephanie PP. Physician Evaluation after Medical Errors: Does Having a Computer Decision Aid Help or Hurt in Hindsight? Medical Decision Making. 2006;26(1):48.

Marsden P. The diagnostic thinking inventory. British journal of hospital medicine. 1992;47(5):389.

Marshall JR, Fleming P, Heffernan M, Kasch S. Pilot study on use of PMPs. Medical education. 1982;16(6):365.

Martin C. The theory of critical thinking of nursing. Nursing Education Perspectives. 2002;23(5):243.

Mary GMT. The Effect of an Educational Model, Developing Nurses' Thinking (DNT), on Nursing Students' Accurate Diagnoses of Patients' Responses to Health Problems. 2011:198 p.

Mathieu S, Couderc M, Glace B, Tournadre A, Malochet-Guinamand S, Pereira B, et al. Construction and utilization of a script concordance test as an assessment tool for DCEM3 (5th year) medical students in rheumatology. BMC Medical Education. 2013;13:166.

Matsell DG, Wolfish NM, Hsu E. Reliability and validity of the objective structured clinical examination in paediatrics. Medical education. 1991;25(4):293.

Mattick K, Dennis I, Bradley P, Bligh J. Content specificity: is it the full story? Statistical modelling of a clinical skills examination. Medical education. 2008;42(6):589.

McBride ME, Waldrop WB, Fehr JJ, Boulet JR, Murray DJ. Simulation in pediatrics: the reliability and validity of a multiscenario assessment. Pediatrics. 2011;128(2):335.

McCallum J, Ness V, Price T. Exploring nursing students' decision-making skills whilst in a Second Life clinical simulation laboratory. Nurse education today. 2011;31(7):699.

McComas MJ, Wright RA, Mann NK, Cooper MD, Jacks ME. M-OSCE as a method to measure dental hygiene students' critical thinking: a pilot study. Journal of dental education. 2013;77(4):485.

McDonald GT, Larsen HD. A system for developing and evaluating the clinical judgment of dental students. Journal of Prosthetic Dentistry. 1985;53(2):265.

McLaughlin K, Coderre S, Mortis G, Mandin H. Expert-type knowledge structure in medical students is associated with increased odds of diagnostic success. Teaching & Learning in Medicine. 2007;19(1):35.

McLaughlin K, Heemskerk L, Herman R, Ainslie M, Rikers RM, Schmidt HG. Initial diagnostic hypotheses bias analytic information processing in non-visual domains. Medical education. 2008;42(5):496.

McLeod PJ. The impact of educational interventions on the reliability of teachers' assessment of student case reports: a controlled trial. Medical education. 1988;22(2):113.

Medio FJ, Morewitz SJ. Four approaches to using patients to teach and evaluate clinical skills of residents, interns, and students. Journal of the American Osteopathic Association. 1992;92(11):1433.

Merrill C, Michael L. Focal Points and Relationships: a Study of Clinical Reasoning. British Journal of Occupational Therapy. 1997;60(2):57.

Meterissian S, Zabolotny B, Gagnon R, Charlin B. Is the script concordance test a valid instrument for assessment of intraoperative decision-making skills? American Journal of Surgery. 2007;193(2):248.

Meterissian SH. A novel method of assessing clinical reasoning in surgical residents. Surgical Innovation. 2006;13(2):115.

Mew MM, Fossey E. Client-centred aspects of clinical reasoning during an initial assessment using the Canadian Occupational Performance Measure. Australian Occupational Therapy Journal. 1996;43(3):155.

Meyerson AT, Wachtel A, Thornton J. Evaluation of a psychiatric clerkship by videotape. American Journal of Psychiatry. 1977;134(8):883.

Michelle B. Interpretive description of clinical judgment within reflective journals of nursing students particiapting in high-fidelity simulation. 2013:260 p.

Miller DA, Sadler JZ, Mohl PC. Critical thinking in preclinical course examinations. Academic Medicine. 1993;68(4):303.

Miller LT, Lee CJ. Gathering and evaluating evidence in clinical decision-making. Journal of Speech-Language Pathology & Audiology. 2004;28(2):97.

Monajemi A, Schmidt HG, Rikers RM. Assessing patient management plans of doctors and medical students: an illness script perspective. Journal of Continuing Education in the Health Professions. 2012;32(1):4.

Moreira J, Bisoffi Z, Narvaez A, Ende JVd. Bayesian clinical reasoning: does intuitive estimation of likelihood ratios on an ordinal scale outperform estimation of sensitivities and specificities? Journal of evaluation in clinical practice. 2008;14(5):934.

Morris BC. Relationships among academic achievement, clinical decision making, critical thinking, work experience, and NCLEX-RN pass status. 1999:109 p.

Morrison S, Free KW. Writing multiple-choice test items that promote and measure critical thinking. Journal of Nursing Education. 2001;40(1):17.

Moulton CA, Regehr G, Mylopoulos M, MacRae HM. Slowing down when you should: a new model of expert judgment. Academic Medicine. 2007;82(10 Suppl):S109.

Mulder SF, Bleijenberg G, Verhagen SC, Stuyt PM, Schijven MP, Tack CJ. Improved competence after a palliative care course for internal medicine residents. Palliative medicine. 2009;23(4):360.

Murphy JI. The use of focused reflection and articulation to promote the development of clinical reasoning. 2001:150 p.

Murphy JI. Using focused reflection and articulation to promote clinical reasoning: An evidence-based teaching strategy. Nursing Education Perspectives. 2004;25(5):226.

Mylopoulos M, Lohfeld L, Norman GR, Dhaliwal G, Eva KW. Renowned physicians' perceptions of expert diagnostic practice. Academic Medicine. 2012;87(10):1413.

Myung SJ, Kang SH, Phyo SR, Shin JS, Park WB. Effect of enhanced analytic reasoning on diagnostic accuracy: a randomized controlled study. Medical teacher. 2013;35(3):248.

Nancy SP. Comparing Information-Gathering Strategies of Medical Students and Physicians in Diagnosing Simulated Medical Cases. Academic Medicine. 1990;65(2):107.

Narayan SM. Heuristic reasoning about uncertainty in a clinical nursing task (Volumes I and II). 1990:506 p.

Narayan SM, Corcoran-Perry S. Line of reasoning as a representation of nurses' clinical decision making. Research in nursing & health. 1997;20(4):353.

Nasca TJ, Gonnella JS, Hojat M, Veloski J, Erdmann JB, Robeson M, et al. Conceptualization and measurement of clinical competence of residents: a brief rating form and its psychometric properties. Medical teacher. 2002;24(3):299.

Neistadt ME. Teaching clinical reasoning as a thinking frame. American Journal of Occupational Therapy. 1998;52(3):221.

Nendaz MR, Bordage G. Promoting diagnostic problem representation. Medical education. 2002;36(8):760.

Nendaz MR, Gut AM, Louis-Simonet M, Perrier A, Vu NV. Bringing explicit insight into cognitive psychology features during clinical reasoning seminars: a prospective, controlled study. Education for Health. 2011;24(1):496.

Nendaz MR, Gut AM, Perrier A, Louis-Simonet M, Blondon-Choa K, Herrmann FR, et al. Brief report: beyond clinical experience: features of data collection and interpretation that contribute to diagnostic accuracy. Journal of General Internal Medicine. 2006;21(12):1302.

Nendaz MR, Gut AM, Perrier A, Reuille O, Louis-Simonet M, Junod AF, et al. Degree of concurrency among experts in data collection and diagnostic hypothesis generation during clinical encounters. Medical education. 2004;38(1):25.

Newble DI, Hoare J, Baxter A. Patient management problems. Issues of validity. Medical education. 1982;16(3):137.

Newble DI, Swanson DB. Psychometric characteristics of the objective structured clinical examination. Medical education. 1988;22(4):325.

Niedringhaus LK. Using student writing assignments to assess critical thinking skills: a holistic approach. Holistic nursing practice. 2001;15(3):9.

Nielsen A, Stragnell S, Jester P. Guide for reflection using the clinical judgment model. Journal of Nursing Education. 2007;46(11):513.

Noble C, Brien MO, Coombes I, Shaw PN, Nissen L. Concept mapping to evaluate an undergraduate pharmacy curriculum. American Journal of Pharmaceutical Education. 2011;75(3):55.

Noguchi Y, Matsui K, Imura H, Kiyota M, Fukui T. A traditionally administered short course failed to improve medical students' diagnostic performance. A quantitative evaluation of diagnostic thinking. Journal of General Internal Medicine. 2004;19(5 Pt 1):427.

Norcini JJ, Swanson DB, Grosso LJ, Shea JA, Webster GD. A comparison of knowledge, synthesis, and clinical judgment. Multiple-choice questions in the assessment of physician competence. Evaluation & the health professions. 1984;7(4):485.

Norcini JJ, Swanson DB, Grosso LJ, Webster GD. Reliability, validity and efficiency of multiple choice question and patient management problem item formats in assessment of clinical competence. Medical education. 1985;19(3):238.

Norman G. Research in clinical reasoning: past history and current trends. Medical education. 2005;39(4):418.

Norman G, Sherbino J, Dore K, Wood T, Young M, Gaissmaier W, et al. The etiology of diagnostic errors: a controlled trial of system 1 versus system 2 reasoning. Academic Medicine. 2014;89(2):277.

Norman GR. Objective measurement of clinical performance. Medical education. 1985;19(1):43.

Norman GR, Barrows HS, Feightner JW, Neufeld VR. Measuring the outcome of clinical problem-solving. Annual Conference on Research in Medical Education. 1977;16:311.

Norman GR, Feightner JW. A comparison of behaviour on simulated patients and patient management problems. Medical education. 1981;15(1):26.

Norman GR, Tugwell P, Feightner JW, Muzzin LJ, Jacoby LL. Knowledge and clinical problem-solving. Medical education. 1985;19(5):344.

Nouh T, Boutros M, Gagnon R, Reid S, Leslie K, Pace D, et al. The script concordance test as a measure of clinical reasoning: a national validation study. American Journal of Surgery. 2012;203(4):530.

Nu Viet V. Medical Problem-Solving Assessment: A Review of Methods and Instruments. Evaluation and the Health Professions. 1979;2(3):281.

Oermann M, Truesdell S, Ziolkowski L. Strategy to assess, develop, and evaluate critical thinking. Journal of continuing education in nursing. 2000;31(4):155.

Oermann MH. Evaluating critical thinking in clinical practice. Nurse educator. 1997;22(5):25.

Offredy M. Decision-making in primary care: outcomes from a study using patient scenarios. Journal of advanced nursing. 2002;40(5):532.

Okubo Y, Ishiguro N, Suganuma T, Nishikawa T, Takubo T, Kojimahara N, et al. Team-based learning, a learning strategy for clinical reasoning, in students with problem-based learning tutorial experiences. Tohoku Journal of Experimental Medicine. 2012;227(1):23.

Pacsi AL. Human simulators in nursing education. Journal of the New York State Nurses Association. 2008;39(2):8.

Page G, Bordage G. The Medical Council of Canada's key features project: a more valid written examination of clinical decision-making skills. Academic Medicine. 1995;70(2):104.

Page G, Bordage G, Allen T. Developing key-feature problems and examinations to assess clinical decision-making skills. Academic Medicine. 1995;70(3):194.

Palchik NS, Wolf FM, Cassidy JT, Ike RW, Davis WK. Case differences in the problem solving strategies of medical students and physicians. Research in medical education:proceedings of the annual Conference. 1988;27:248.

Palmer EJ, Duggan P, Devitt PG, Russell R. The modified essay question: its exit from the exit examination? Medical teacher. 2010;32(7):e300.

Panzarella KJ, Manyon AT. A model for integrated assessment of clinical competence. Journal of allied health. 2007;36(3):157.

Papp KK, Williams SD, Goldman MH. Relationship between type of surgical clerkship, order of completion, and achievement on patient management problems. Surgery. 1984;96(1):102.

Pardamean B. Measuring change in critical thinking skills of dental students educated in a PBL curriculum. Journal of dental education. 2012;76(4):443.

Park AJ, Barber MD, Bent AE, Dooley YT, Dancz C, Sutkin G, et al. Assessment of intraoperative judgment during gynecologic surgery using the Script Concordance Test. American Journal of Obstetrics & Gynecology. 2010;203(3):240.e1.

Patricia B. Using the Dreyfus Model of Skill Acquisition to Describe and Interpret Skill Acquisition and Clinical Judgment in Nursing Practice and Education. Bulletin of Science, Technology & Society. 2004;24(3):188.

Paul B, Gerry H. Assessing the ability of medical students to apply evidence in practice: The potential of the OSCE. Medical education. 1999;33(11):815.

Paul RB. Involving Program Beneficiaries in the Early Stages of Evaluation: Issues of Consequential Validity and Influence. Educational Evaluation and Policy Analysis. 1993:420.

Payton OD. Clinical reasoning process in physical therapy. Physical Therapy. 1985;65(6):924.

Pepa CA, Brown JM, Alverson EM. A comparison of critical thinking abilities between accelerated and traditional baccalaureate nursing students. Journal of Nursing Education. 1997;36(1):46.

Petra G. Robot decisions: On the importance of virtuous judgment in clinical decision making. Journal of evaluation in clinical practice. 2011;17(5):883.

Petrucci AM, Nouh T, Boutros M, Gagnon R, Meterissian SH. Assessing clinical judgment using the Script Concordance test: the importance of using specialty-specific experts to develop the scoring key. American Journal of Surgery. 2013;205(2):137.

Pintz C. Assessment of diagnostic reasoning with standardized patients: testing the reliability and validity of the diagnostic reasoning assessment. 2006:115 p.

Potter BK, Reilly NO, Etchegary H, Howley H, Graham ID, Walker M, et al. Exploring informed choice in the context of prenatal testing: Findings from a qualitative study. Health Expectations. 2008;11(4):355.

Pottier P, Dejoie T, Hardouin JB, Loupp AGL, Planchon B, Bonnaud A, et al. Effect of stress on clinical reasoning during simulated ambulatory consultations. Medical teacher. 2013;35(6):472.

Pottier P, Hardouin JB, Hodges BD, Pistorius MA, Connault J, Durant C, et al. Exploring how students think: a new method combining think-aloud and concept mapping protocols. Medical education. 2010;44(9):926.

Ramezani-Badr F, Nasrabadi AN, Yekta ZP, Taleghani F. Strategies and criteria for clinical decision making in critical care nurses: a qualitative study. Journal of Nursing Scholarship. 2009;41(4):351.

Ramnarayan P, Kapoor RR, Coren M, Nanduri V, Tomlinson AL, Taylor PM, et al. Measuring the impact of diagnostic decision support on the quality of clinical decision making: development of a reliable and valid composite score. Journal of the American Medical Informatics Association. 2003;10(6):563.

Razzouk E, Cohen T, Almoosa K, Patel V. Approaching the limits of knowledge: the influence of priming on error detection in simulated clinical rounds. AMIA Annual Symposium Proceedings/AMIA Symposium. 2011;2011:1155.

Redding DA. The development of critical thinking among students in baccalaureate nursing education. Holistic nursing practice. 2001;15(4):57.

Rego P, Peterson R, Callaway L, Ward M, Brien CO, Donald K. Using a structured clinical coaching program to improve clinical skills training and assessment, as well as teachers' and students' satisfaction. Medical teacher. 2009;31(12):e586.

Reyna VF, Lloyd FJ. Physician decision making and cardiac risk: effects of knowledge, risk perception, risk tolerance, and fuzzy processing. Journal of Experimental Psychology: Applied. 2006;12(3):179.

Reynolds A. Patho-flow diagramming: a strategy for critical thinking and clinical decision making. Journal of Nursing Education. 1994;33(7):333.

Richard OD, Edward HS. Expert Systems Research. Science. 1983;220(4594):261.

Robb Y, Fleming V, Dietert C. Measurement of clinical performance of nurses: a literature review. Nurse education today. 2002;22(4):293.

Robertson J, Walkom E, Pearson SA, Hains I, Williamsone M, Newby D. The impact of pharmacy computerised clinical decision support on prescribing, clinical and patient outcomes: a systematic review of the literature. International Journal of Pharmacy Practice. 2010;18(2):69.

Rochelle JSL, Durning SJ, Pangaro LN, Artino AR, Vleuten CPvd, Schuwirth L. Authenticity of instruction and student performance: a prospective randomised trial. Medical education. 2011;45(8):807.

Rochmawati E, Wiechula R. Education strategies to foster health professional students' clinical reasoning skills. Nursing & health sciences. 2010;12(2):244.

Rock DL, Bransford JD. An empirical evaluation of three components of the tetrahedron model of clinical judgment. Journal of Nervous & Mental Disease. 1992;180(9):560.

Romeo EM. Quantitative research on critical thinking and predicting nursing students' NCLEX-RN performance. Journal of Nursing Education. 2010;49(7):378.

Ronald MC. Place Matters in Physician Practice and Learning. Journal of Continuing Education in the Health Professions. 2003;23.

Ronald ME, Daniel JS, Jordan S. Self-Monitoring in Clinical Practice: A Challenge for Medical Educators. Journal of Continuing Education in the Health Professions. 2008;28(1):5.

Ronald PD, Ruth-Marie EF, Reigart JR, Carol JL, Leonard SL. Association between third-year medical students' abilities to organize hypotheses about patients' problems and to order appropriate diagnostic tests. Academic Medicine. 1991;66(11):702.

Ross PT, Uijtdehaage S, Lypson ML. Reflections on culture: views on script concordance testing. Medical education. 2010;44(5):505.

Round AP. Teaching clinical reasoning--a preliminary controlled study. Medical education. 1999;33(7):480.

Roy RB, McMahon GT. Video-based cases disrupt deep critical thinking in problem-based learning. Medical education. 2012;46(4):426.

Ruland C. Clinical decision making in nursing. Nordic Journal of Nursing Research & Clinical Studies / Vård i Norden. 1996;16(4):4.

Ruthman J, Jackson J, Cluskey M, Flannigan P, Folse VN, Bunten J. Using clinical journaling to capture critical thinking across the curriculum. Nursing Education Perspectives. 2004;25(3):120.

Sacchi S, Cherubini P. The effect of outcome information on doctors' evaluations of their own diagnostic decisions. Medical education. 2004;38(10):1028.

Saintsing D, Gibson LM, Pennington AW. The novice nurse and clinical decision-making: how to avoid errors. Journal of nursing management. 2011;19(3):354.

Salsali M, Tajvidi M, Ghiyasvandian S. Critical thinking dispositions of nursing students in Asian and non-Asian countries: a literature review. Global Journal of Health Science. 2013;5(6):172.

Schell BA. Clinical reasoning and occupation-based practice: changing habits. OT Practice. 2003;8(18):CE-1-CE-8, 2p.

Schmidt HG, Norman GR, Boshuizen HP. A cognitive perspective on medical expertise: theory and implication. Academic Medicine. 1990;65(10):611.

Schriver AT, Morrow DG, Wickens CD, Talleur DA. Expertise differences in attentional strategies related to pilot decision making. Human factors. 2008;50(6):864.

Schubert A, Tetzlaff JE, Tan M, Ryckman JV, Mascha E. Consistency, inter-rater reliability, and validity of 441 consecutive mock oral examinations in anesthesiology: implications for use as a tool for assessment of residents. Anesthesiology. 1999;91(1):288.

Schuwirth L. Is assessment of clinical reasoning still the Holy Grail? Medical education. 2009;43(4):298.

Schweitzer TA. Thought processes and factors influencing recently graduated registered nurses' clinical reasoning. 2008:204

Scott LD, Arslanian-Engoren C, Engoren MC. Association of sleep and fatigue with decision regret among critical care nurses. American Journal of Critical Care. 2014;23(1):13-23.

Scott JN, Markert RJ, Dunn MM. Critical thinking: change during medical school and relationship to performance in clinical clerkships. Medical education. 1998;32(1):14.

Scott-Smith W. The development of reasoning skills and expertise in primary care. Education for Primary Care. 2006;17(2):117.

Seldomridge EA. The influence of confidence, factual, and experiential knowledge on speed and accuracy of clinical judgment among novice and expert nurses. 1996:180 p.

Setna Z, Jha V, Boursicot KA, Roberts TE. Evaluating the utility of workplace-based assessment tools for speciality training. Best Practice & Research in Clinical Obstetrics & Gynaecology. 2010;24(6):767.

Shaban RZ. Theories of clinical judgment and decision-making: a review of the theoretical literature. Journal of Emergency Primary Health Care. 2005;3(1):13p.

Shallaly GEE, Mekki AM. Use of computer-based clinical examination to assess medical students in surgery. Education for Health. 2012;25(3):148.

Sherbino J, Dore KL, Wood TJ, Young ME, Gaissmaier W, Kreuger S, et al. The relationship between response time and diagnostic accuracy. Academic Medicine. 2012;87(6):785.

Sherbino J, Yip S, Dore KL, Siu E, Norman GR. The effectiveness of cognitive forcing strategies to decrease diagnostic error: an exploratory study. Teaching & Learning in Medicine. 2011;23(1):78.

Siassakos D, Draycott TJ, Crofts JF, Hunt LP, Winter C, Fox R. More to teamwork than knowledge, skill and attitude. BJOG: An International Journal of Obstetrics & Gynaecology. 2010;117(10):1262.

Sibbald M, Bruin ABd, Merrienboer JJv. Checklists improve experts' diagnostic decisions. Medical education. 2013;47(3):301.

Sibbald M, Cavalcanti RB. The biasing effect of clinical history on physical examination diagnostic accuracy. Medical education. 2011;45(8):827.

Sibbald M, Panisko D, Cavalcanti RB. Role of clinical context in residents' physical examination diagnostic accuracy. Medical education. 2011;45(4):415.

Sibert L, Charlin B, Corcos J, Gagnon R, Grise P, Vleuten Cvd. Stability of clinical reasoning assessment results with the Script Concordance test across two different linguistic, cultural and learning environments. Medical teacher. 2002;24(5):522.

Simpson RG, Ballard KD. What is being assessed in the MRCGP oral examination? A qualitative study. British Journal of General Practice. 2005;55(515):430.

Sinatra-Wilhelm T. Nursing care plans versus concept maps in the enhancement of critical thinking skills in nursing students enrolled in a baccalaureate nursing program. Creative nursing. 2012;18(2):78.

Sinclair K. A model for the development of clinical reasoning in occupational therapy. 2003:365 p.

Singleton J, Levin R. Strategies for learning evidence-based practice: critically appraising clinical practice guidelines. Journal of Nursing Education. 2008;47(8):380.

Sladyk KA. Clinical reasoning and reflective practice: influence of fieldwork activities. 1997:280 p.

Slater SC, Boulet JR. Predicting holistic ratings of written performance assessments from analytic scoring. Advances in Health Sciences Education. 2001;6(2):103.

Smith A. Measuring the use of intuition by registered nurses in clinical practice. Nursing Standard. 2007;21(47):35.

Smith AJ, Thurkettle MA, Cruz FAd. Use of intuition by nursing students: instrument development and testing. Journal of advanced nursing. 2004;47(6):614.

Smith CS. A developmental approach to evaluating competence in clinical reasoning. Journal of Veterinary Medical Education. 2008;35(3):375.

Smith RM. The triple-jump examination as an assessment tool in the problem-based medical curriculum at the University of Hawaii. Academic Medicine. 1993;68(5):366.

Sobral DT. Medical students' mindset for reflective learning: a revalidation study of the reflection-in-learning scale. Advances in Health Sciences Education. 2005;10(4):303.

Solomon DJ, Laird-Fick HS, Keefe CW, Thompson ME, Noel MM. Using a formative simulated patient exercise for curriculum evaluation. BMC Medical Education. 2004;4:8.

Souza TA. Back to basics: an overview of clinical reasoning and problem solving. Topics in Clinical Chiropractic. 1998;5(2):1.

Spake EF. Clinical reasoning and decision making of experienced clinicians and entry-level physical therapist students. 2003:143 p.

Spelic SS, Parsons M, Hercinger M, Andrews A, Parks J, Norris J. Evaluation of critical thinking outcomes of a BSN program. Holistic nursing practice. 2001;15(3):27.

Steinemann SK, Berg BW, Turban JW, Hara KM, Alfrey LR. Student-written simulation scenarios: a novel cognitive assessment method in a trauma curriculum. Hawaii medical journal. 2011;70(8):172.

Stieger S, Praschinger A, Kletter K, Kainberger F. Diagnostic grand rounds: a new teaching concept to train diagnostic reasoning. European Journal of Radiology. 2011;78(3):349.

Stiegler MP, Neelankavil JP, Canales C, Dhillon A. Cognitive errors detected in anaesthesiology: a literature review and pilot study. British journal of anaesthesia. 2012;108(2):229.

Stiegler MP, Ruskin KJ. Decision-making and safety in anesthesiology. Current Opinion in Anaesthesiology. 2012;25(6):724.

Stone CA, Davidson LJ, Evans JL, Hansen MA. Validity evidence for using a general critical thinking test to measure nursing students' critical thinking. Holistic nursing practice. 2001;15(4):65.

Stover LM. The relationship between perceived clinical decision making ability and medication dosage calculation ability of registered nurses. 2000:92 p.

Struchiner M, Vieira AR, Ricciardi RM. [Analysis of dental students' knowledge and concepts in oral health: evaluation by concept maps]. Cadernos de Saude Publica. 1999;15(Suppl 2):55.

Stubbings L, Chaboyer W, McMurray A. Nurses' use of situation awareness in decision-making: an integrative review. Journal of advanced nursing. 2012;68(7):1443.

Szatmary P, Arora S, Sevdalis N. To operate or not to operate? A multi-method analysis of decision-making in emergency surgery. American Journal of Surgery. 2010;200(2):298.

Tang B, Hanna GB, Carter F, Adamson GD, Martindale JP, Cuschieri A. Competence assessment of laparoscopic operative and cognitive skills: Objective Structured Clinical Examination (OSCE) or Observational Clinical Human Reliability Assessment (OCHRA). World journal of surgery. 2006;30(4):527.

Tanner CA. Thinking like a nurse: a research-based model of clinical judgment in nursing. Journal of Nursing Education. 2006;45(6):204.

Taylor C. Clinical problem-solving in nursing: insights from the literature. Journal of advanced nursing. 2000;31(4):842.

Taylor TR, Skakun EN, Taylor WC. The assessment of competence in clinical decision-making: a computer approach based on decision theory. Journal of medical education. 1976;51(8):687.

Tesoro MG. Effects of using the developing nurses' thinking model on nursing students' diagnostic accuracy. Journal of Nursing Education. 2012;51(8):436.

Teunissen PW, Stapel DA, Scheele F, Scherpbier AJ, Boor K, Diemen-Steenvoorde JAv, et al. The influence of context on residents' evaluations: effects of priming on clinical judgment and affect. Advances in Health Sciences Education. 2009;14(1):23.

Thompson C, Dalgleish L, Bucknall T, Estabrooks C, Hutchinson AM, Fraser K, et al. The effects of time pressure and experience on nurses' risk assessment decisions: a signal detection analysis. Nursing research. 2008;57(5):302.

Thompson CAB. The cognitive structure of clinical expertise. 1992:233 p.

Thorpe K, Loo R. Critical-thinking types among nursing and management undergraduates. Nurse education today. 2003;23(8):566.

Tona JT. Variations in clinical reasoning among occupational therapy practitioners. 2003:210 p.

Torcivia EM. The development of a matrix for the evaluation of clinical reasoning in occupational therapists. 2007:325 p.

Tornyay RD. Measuring problem-solving skills by means of the simulated clinical nursing problem test. Journal of Nursing Education. 1968;7(3):3.

Torre DM, Daley B, Stark-Schweitzer T, Siddartha S, Petkova J, Ziebert M. A qualitative evaluation of medical student learning with concept maps. Medical teacher. 2007;29(9):949.

Vachon B, LeBlanc J. Effectiveness of past and current critical incident analysis on reflective learning and practice change. Medical education. 2011;45(9):894.

Vadali M, Ramachandran G, Banerjee S. Effect of training, education, professional experience, and need for cognition on accuracy of exposure assessment decision-making. Annals of Occupational Hygiene. 2012;56(3):292.

Verkoeijen PP, Rikers RM, Schmidt HG, Wiel MWvd, Kooman JP. Case representation by medical experts, intermediates and novices for laboratory data presented with or without a clinical context. Medical education. 2004;38(6):617.

Vernon RC, Roger B, Pauline D, William HE, Scott MM, Greg PS, et al. Effectiveness of a Simulated Clinical Examination in the Assessment of the Clinical Competencies of Entry-Level Trainees in a Family Medicine Residency Programme. Assessment & Evaluation in Higher Education. 2012;37(1):99.

Vicki RL, Lee RB, Geoffrey RN. Believing Is Seeing: The Influence of a Diagnostic Hypothesis on the Interpretation of Clinical Features. Academic Medicine. 2002;77(10):S67.

Victor-Chmil J. Critical thinking versus clinical reasoning versus clinical judgment: differential diagnosis. Nurse educator. 2013;38(1):34.

Victoria M. Exploring Indigenous health using the clinical reasoning cycle: A student paper. Contemporary Nurse: A Journal for the Australian Nursing Profession. 2010;37(1):82.

Vito-Thomas PAD. The relationship between nursing student performance and critical thinking in clinical judgment. 2002:124 p.

Vito-Thomas PD. Identifying critical thinking behaviors in clinical judgments. Journal for Nurses in Staff Development - JNSD. 2000;16(4):174.

Vleuten CPvd, Newble DI. How can we test clinical reasoning? Lancet. 1995;345(8956):1032.

Voytko DC. A comparison of the cognitive processes used by the novice and the expert nurse in making clinical decisions. 1989:191 p.

Vries TPD. Presenting clinical pharmacology and therapeutics: the course in pharmacotherapeutics. British journal of clinical pharmacology. 1993;35(6):587.

Vu NV, Paiva RE, Dawson-Saunders BK, Marcy ML, Barrows HS. Incremental validity of the Medical Reasoning Aptitude Test (MRAT)--a new admission test of clinical problem solving ability. Proceedings of the Annual Conference on Research in Medical Education. 1983;22:61.

Wainwright SF. Novice and experienced physical therapy clinicians: a comparison of clinical decision making abilities. 2006:380 p.

Weatherspoon DL, Wyatt TH. Testing computer-based simulation to enhance clinical judgment skills in senior nursing students. Nursing Clinics of North America. 2012;47(4):481.

Wessel J, Williams R, Cole B. Physical therapy students' application of a clinical decision-making model. Internet Journal of Allied Health Sciences & Practice. 2006;4(3):11p.

West DC, Pomeroy JR, Park JK, Gerstenberger EA, Sandoval J. Critical thinking in graduate medical education: A role for concept mapping assessment? JAMA. 2000;284(9):1105.

Wetmore AO, Boyd LD, Bowen DM, Pattillo RE. Reflective blogs in clinical education to promote critical thinking in dental hygiene students. Journal of dental education. 2010;74(12):1337.

Wheeler LA, Collins SK. The influence of concept mapping on critical thinking in baccalaureate nursing students. Journal of Professional Nursing. 2003;19(6):339.

William CC, Ellen FC. Psychology of the scientist: LVII. Can humanistic theory contribute to our understanding of medical problem-solving? Psychological reports. 1987;61(3):779.

William CM. Medical Problem-Solving: A Reanalysis. Journal of medical education. 1980;55(11):912.

Wong TK, Chung JW. Diagnostic reasoning processes using patient simulation in different learning environments. Journal of Clinical Nursing. 2002;11(1):65.

Wood TJ, Cunnington JP, Norman GR. Assessing the measurement properties of a clinical reasoning exercise. Teaching & Learning in Medicine. 2000;12(4):196.

Woods NN, Brooks LR, Norman GR. The role of biomedical knowledge in diagnosis of difficult clinical cases. Advances in Health Sciences Education. 2007;12(4):417.

Worrell JA, Profetto-McGrath J. Critical thinking as an outcome of context-based learning among post RN students: a literature review. Nurse education today. 2007;27(5):420.

Yalof J, Abraham P. An integrative approach to assessment supervision. Bulletin of the Menninger Clinic. 2009;73(3):188.

Yang RL, Hashimoto DA, Predina JD, Bowens NM, Sonnenberg EM, Cleveland EC, et al. The virtual-patient pilot: testing a new tool for undergraduate surgical education and assessment. Journal of Surgical Education. 2013;70(3):394.

Yaphe J, Street S. How do examiners decide?: a qualitative study of the process of decision making in the oral examination component of the MRCGP examination. Medical education. 2003;37(9):764.

Young ME, Brooks LR, Norman GR. The influence of familiar non-diagnostic information on the diagnostic decisions of novices. Medical education. 2011;45(4):407.

Yurko YY, Scerbo MW, Prabhu AS, Acker CE, Stefanidis D. Higher mental workload is associated with poorer laparoscopic performance as measured by the NASA-TLX tool. Simulation in Healthcare: The Journal of The Society for Medical Simulation. 2010;5(5):267.

Zwaan L, Thijs A, Wagner C, Wal Gvd, Timmermans DR. Relating faults in diagnostic reasoning with diagnostic errors and patient harm. Academic Medicine. 2012;8
